# Nuclear and cytoplasmic WDR-23 isoforms mediate differential effects on GEN-1 and SKN-1 substrates

**DOI:** 10.1038/s41598-019-48286-y

**Published:** 2019-08-13

**Authors:** Brett N. Spatola, Jacqueline Y. Lo, Bin Wang, Sean P. Curran

**Affiliations:** 1 University of Southern California, Leonard Davis School of Gerontology, Los Angeles, CA USA; 2 University of Southern California, Molecular and Computational Biology, Los Angeles, CA USA; 3 Present Address: Buck Institute for Aging Research, Novato, CA USA; 4 The University of Dundee, Centre for Gene Expression and Regulation, Dundee, Scotland; 5 University of Southern California, Norris Comprehensive Cancer Center, Los Angeles, CA USA

**Keywords:** Ubiquitylation, Genetics, Molecular biology, Ubiquitylation, Transcription factors

## Abstract

Maintaining a healthy cellular environment requires the constant control of proteostasis. E3 ubiquitin ligase complexes facilitate the post-translational addition of ubiquitin, which based on the quantity and specific lysine linkages, results in different outcomes. Our studies reveal the CUL4-DDB1 substrate receptor, WDR23, as both a positive and a negative regulator in cellular stress responses. These opposing roles are mediated by two distinct isoforms: WDR-23A in the cytoplasm and WDR-23B in the nucleus. *C. elegans* expressing only WDR-23A display activation of SKN-1 and enhanced survival to oxidative stress, whereas animals with restricted WDR-23B expression do not. Additionally, we identify GEN-1, a Holliday junction resolvase, as an evolutionarily conserved WDR-23 substrate and find that the nuclear and cytoplasmic isoforms of WDR-23 differentially affect double-strand break repair. Our results suggest that through differential ubiquitination, nuclear WDR-23B inhibits the activity of substrates, most likely by promoting protein turnover, while cytoplasmic WDR-23A performs a proteasome-independent role. Together, our results establish a cooperative role between two spatially distinct isoforms of WDR-23 in ensuring proper regulation of WDR-23 substrates.

## Introduction

Proteostasis plays an integral part in ensuring organismal survival, and numerous diseases can arise when the balance of the creation, maintenance, and degradation of proteins is dysregulated^1,2^. A major component of proteostasis is the ubiquitin-proteasome system (UPS), which is the primary cellular degradation pathway. The most critical player in the UPS is the E3 ubiquitin ligase complex, which assists in adding ubiquitin onto substrates *via* concurrently binding the substrates and the ubiquitin bound-E2 conjugation enzyme. Additional ubiquitins are attached *via* K48 linkage in order for the substrate to be presented to the proteasome for degradation. Interestingly, the quantity of ubiquitins added and the type of inter-ubiquitin linkage can have different outcomes; the attachment of multi- and/or mono-ubiquitination on proteins can trigger activation, translocation, and other signaling cascades that can be beneficial to the cell^3,4^.

Over 600 E3 ubiquitin ligases have been identified in humans^4,5^. One of the most abundant, yet complex, group of E3 ubiquitin ligases are the cullin-RING ligases (CRLs), which form multi-protein complexes. Cullins serve as the main platform of the CRL complex^6^, with the C-terminal portion bound by RING finger proteins and the N-terminal domain bound to adaptor proteins, such as DDB1. Adaptor proteins form the bridge between the cullin and the substrate receptor, which directly bind to substrates. Substrate receptors, or DDB1-CUL4 associated factors (DCAFs), are an important part of the CRL, but there is a gap in the identification of specific substrates^7^. Approximately 90 receptors have been discovered^8^, but only a fraction of these have been associated with specific substrates^9–14^. As many identified substrates have been linked in some form to cancer^15^, this places CRLs at the forefront of chemotherapeutic and pharmacological treatments.

WDR-23, the *C. elegans* homolog of mammalian DCAF11/WDR23, is a CRL substrate receptor that exists in two spatially distinct isoforms, a cytoplasmic WDR-23A and nuclear WDR-23B^16–18^. Identification of direct substrates for WDR23 are emerging^17,19,20^, but the interplay and specific functions of each have not been well-studied. One previous study observed that extra-chromosomal expression of *wdr-23A* cDNA, but not *wdr-23B* cDNA, in *wdr-23(k1007)* mutants suppresses fluorescence of a target gene of a known substrate that is negatively regulated by WDR-23, SKN-1^18^. However, the mosaic nature of extra-chromosomal arrays, their high copy number, their silencing in some tissues like the germline, and/or the nature of the *wdr-23(k1007)* variant used in this study alone or in combination likely impact WDR-23 and/or SKN-1 function. As such, a thorough examination on the effect of stable and physiological rescue of the cytoplasmic (*wdr-23A)* or nuclear (*wdr-23B)* isoforms in a *wdr-23* mutant on SKN-1 and other substrates activity has not been pursued.

More recently, our lab discovered that NRF2 is a substrate of DCAF11^17^. NRF2, the human ortholog of SKN-1 in *C. elegans*, is a transcription factor that activates cytoprotective genes upon cellular stress^21,22^. Results from this study and others highlight DCAF11’s role in the degradation of proteins, organismal health, and even lifespan^17,19,23^. However, the possibility of proteasome-independent functions have been documented for DCAF11, as DCAF11 was found to ensure histone supply during DNA replication *via* ubiquitination of its substrate SLBP (stem-loop binding protein)^20^. Additional studies must be conducted to further examine the diverse roles of WDR23 in protein regulation.

With a combination of *C. elegans* genetics and mammalian cell culture, we present the spatially distinct mechanism of WDR-23-mediated regulation in two cellular pathways and identify GEN-1 as a new substrate of this CRL complex.

## Results

### Nuclear WDR-23B, but not cytoplasmic WDR-23A, negatively regulates oxidative stress resistance

**Figure 1:**
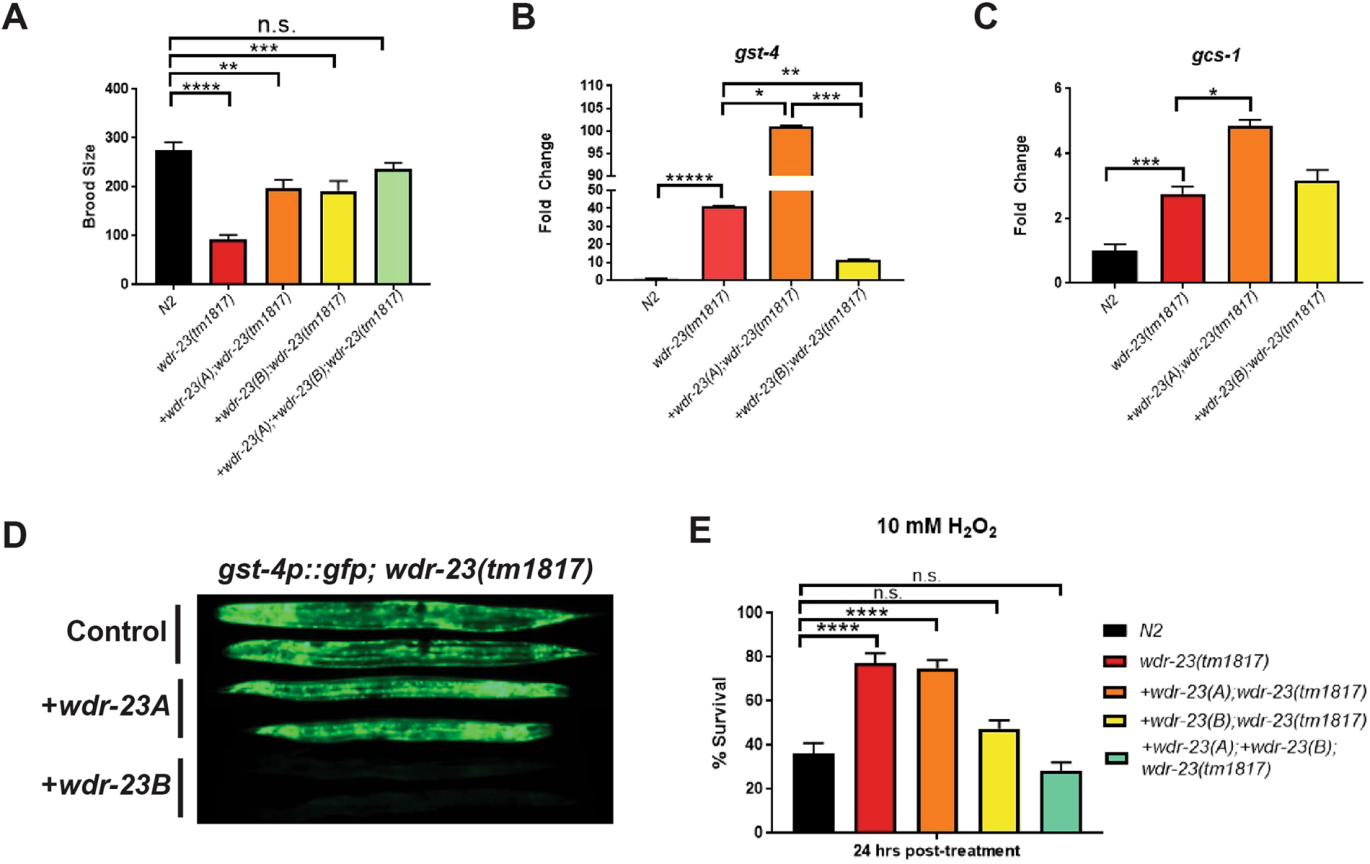
Nuclear WDR-23B negatively impacts SKN-1 activity. (**A**) Defects in total brood size in the *wdr-23(tm1817)* null mutant are partially restored in *wdr-23(tm1817);* +*wdr-23(A)* and *wdr-23(tm1817);* +*wdr-23(B)* animals while expression of both WDR-23A and WDR-23B restores brood size to wild-type levels; n ≥ 10 animals for each strain. SKN-1 target gene (**B**) *gst-4* is significantly decreased in +*wdr-23(B); wdr-23(tm1817)* compared to +*wdr-23(A); wdr-23(tm1817)* and *wdr-23(tm1817)* animals. (**C**) SKN-1 target gene *gcs-1* is significantly higher in +*wdr-23(A); wdr-23(tm1817)* compared to *wdr-23(tm1817)* worms. n = 5 plates of ~1000 animals for each strain. (**D**) Representative images of *gst-4p::gfp* expression in control (*wdr-23(tm1817))*, +*wdr-23(A); wdr-23(tm1817)*, and +*wdr-23(B); wdr-23(tm1817)* animals. Images were taken at 20 ms in L4 or later worms. (**E**) Decreased survival percentage in +*wdr-23(B); wdr-23(tm1817)* when compared to *wdr-23(tm1817)* and +*wdr-23(A); wdr-23(tm1817)* animals after exposure to 10 mM H_2_O_2_. n ≥ 10 plates of ≥10 animals for each strain. Data are mean ± s.e.m.; one-way ANOVA with multiple comparisons. n.s. = not significant *p < 0.05,**p < 0.01, ***p < 0.001, ****p < 0.0001, *****p < 0.00001. (See Table S1 for more details).

To dissect the differential roles of the nuclear and cytoplasmic isoforms of WDR-23, we utilized a *wdr-23(tm1817)* null mutant where WDR-23 is rescued with integrated single-copy transgenes of cytoplasmic *wdr-23a* cDNA or nuclear *wdr-23b* cDNA^16^ (Fig. 1A). The null mutant *wdr-23(tm1817)* contains a 635 bp deletion and is known to have an extreme defect in fecundity^24^; thus, we tested for the capacity of each isoform to restore brood size back to wild type levels. Although rescue from either WDR-23 isoform increased brood size, neither was able to fully restore fertility (Fig. 1A), suggesting that both isoforms are needed for normal reproductive output and importantly, that the nuclear and cytoplasmic versions of WDR-23 have distinct roles. Indeed, restoration of both WDR-23A and WDR-23B in *wdr-23(tm1817)* mutants fully restored brood size to wild type levels (Fig. 1A).

We next investigated if WDR-23 isoforms differentially regulate SKN-1, a known substrate for WDR-23. SKN-1, or NRF2 in mammals, is a cytoprotective transcription factor that activates upon cellular stresses, such as oxidative stress^25^. WDR-23 has been shown to negatively regulate SKN-1 stability; the null mutant *wdr-23(tm1817)* has increased SKN-1 protein levels, resulting in an increase in oxidative stress resistance when compared to wild type worms^21^. To determine the role of each isoform, we first measured transcript levels of SKN-1 target genes (*gst-4* and *gcs-1*) *via* qPCR in the strains with isoform-specific rescue of WDR-23 (Fig. 1B,C). Consistent with previous data^21^, *wdr-23(tm1817)* showed a significant increase (~40-fold) in *gst-4* mRNA levels (Fig. 1B). Interestingly, we observed that *gst-4* mRNA levels were further increased, to a level of ~100 fold greater than wild type animals, when only the cytoplasmic WDR-23A isoform is restored (Fig. 1B). The same synergistic effect was observed when studying mRNA levels of *gcs-1* (Fig. 1C). The increased expression of SKN-1 target genes in strains solely expressing the cytoplasmic WDR-23 isoform suggests a positive role for WDR-23A on SKN-1 activity, a surprising finding given the canonical role of WDR-23 mediating the proteasomal turnover of SKN-1. In contrast, expression of the nuclear WDR-23B isoform reduced *gst-4* mRNA levels as compared to the *wdr-23(tm1817)* mutant (Fig. 1B), a result expected if the primary function of nuclear WDR-23B is to stimulate SKN-1 turnover. The expression of *gcs-1* was not restored in animals with expression of only WDR-23B; however, it is notable that the impact of SKN-1 activation on *gst-4* and *gcs-1* differs by an order of magnitude, perhaps indicating differences in the regulatory pathways that govern these genes beyond SKN-1. The isoform specific roles of WDR-23 *on gst-4* activation were corroborated *in vivo* by measuring GFP expression from a SKN-1 target gene reporter, *gst-4p::gfp*, in combination with each one of the WDR-23 isoforms rescuing *wdr-23(tm1817)*, where we observed a significant decrease of GFP fluorescence only in WDR-23B rescue worms (Figs 1D; S1A,B). Furthermore, gDNA rescue of both WDR-23A and WDR-23B in *wdr-23(tm1817)* mutants fully restored *gst-4::gfp* expression levels to wild type levels (Figs 1D, S1A,B).

Based on the opposing expression levels of SKN-1 target gene mRNAs upon expression of the nuclear or cytoplasmic forms of WDR-23, we wondered if these animals would display differential resistance to oxidative stress. To test this, worms were challenged with an acute exposure to hydrogen peroxide (H_2_O_2_), and survival rates were measured 24 hours later^17,26,27^. Consistent with previous data^21,23^, *wdr-23(tm1817)* displayed enhanced survival compared to wild type, a phenotype only suppressed by WDR-23B expression (Fig. 1E), further suggesting the negative impact on the activity of SKN-1 by WDR-23B. WDR-23A expressing worms remained resistant to oxidative stress to the same degree as the *wdr-23(tm1817)* (Fig. 1E). Furthermore, gDNA rescue of both WDR-23A and WDR-23B in *wdr-23(tm1817)* mutants fully restored survival levels to wild type levels (Fig. 1E).

In summary, these results suggest the opposing functions of WDR-23 on SKN-1 activity is based on the cellular localization of each isoform. Since the phenotypes stemming from the restoration of both WDR-23A and WDR-23B resembles the rescue of WDR-23B alone, we suggest that WDR-23B activity follows the actions of WDR-23A.

### WDR-23 interacts with GEN-1

**Figure 2:**
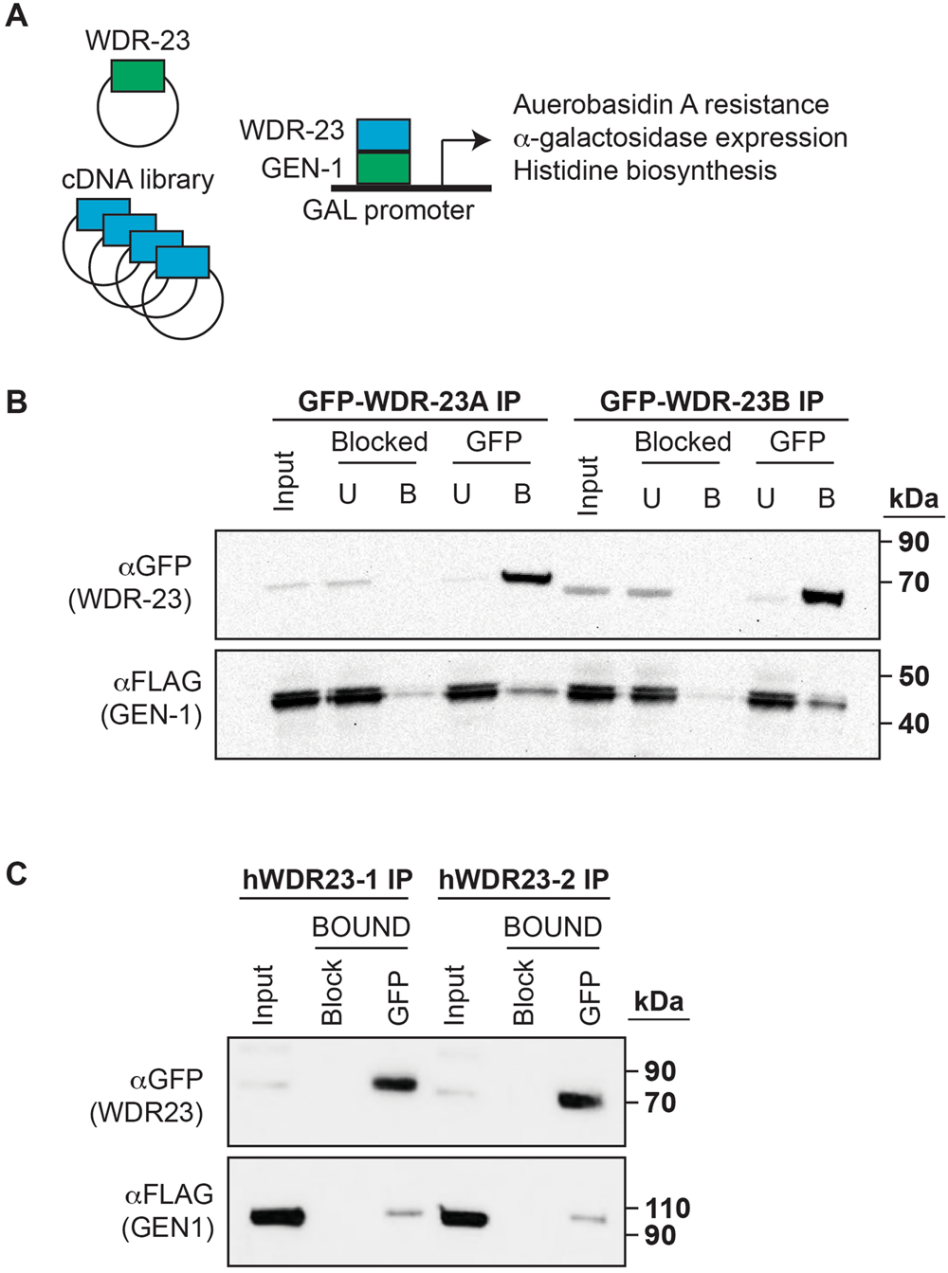
WDR-23 interacts with GEN-1. (**A**) Identification of *gen-1* as a potential interactor with *wdr-23* through a yeast-2-hybrid screen. (**B**) GEN-1 co-immunoprecipitates with both WDR-23 isoforms in HEK293T cells overexpressing tagged worm versions of GEN-1-FLAG and GFP-WDR-23. (**C**) The interaction between GEN1 and both isoforms of WDR23 is conserved in HEK293T cells overexpressing tagged human versions of FLAG-GEN1 and GFP-WDR23. Blocked = blocked magnetic agarose GFP beads. GFP = magnetic agarose GFP beads. Input = 2.5% of whole-cell extract used for IP. U = unbound/flow through fraction. B = bound fraction. αGFP probed for GFP-WDR-23/GFP-WDR23 isoforms. αFLAG for GEN-1-FLAG/FLAG-GEN1.

Given the unexpected finding that WDR-23 isoforms may play opposing roles in the regulation of SKN-1 activity, we sought to identify new substrates for WDR-23. We conducted a yeast two-hybrid screen, using *wdr-23A* as bait and a *C. elegans* cDNA library as prey^28^ (Fig. 2A). From a collection of several candidate proteins, we identified GEN-1 (GEN1 in humans) as a potential interactor (Table S2). GEN1 functions as a conserved Holliday junction resolvase and participates in DNA damage signaling pathways across species^29,30^. Our previous studies revealed that overexpression of human WDR23 in HEK293T cells decreased cell viability and increased accumulation of DNA double strand breaks (DSBs) after exposure to DSB-inducing chemotherapeutic drugs^17^. Although these studies focused on WDR23-mediated NRF2 stability, the possibility that WDR23 has other targets that could impact genome stability remained plausible. The regulation of GEN1 must be tightly controlled to avoid chromosomal abnormalities; yeast and human cells have developed measures to restrict GEN1 localization away from the nucleus, thus prohibiting activity on chromatin, *via* phosphorylation and a nuclear export signal, respectively^31,32^. Taken together, the activity of GEN1 is regulated by its subcellular localization, and WDR-23 could potentially mediate this process.

To biochemically confirm the physical interaction between WDR-23 and GEN-1, we transiently expressed GFP- and FLAG-tagged worm WDR-23 isoforms and GEN-1, respectively, in HEK293T cells and performed co-IP (co-immunoprecipitation). We observed an enrichment of GEN-1-FLAG protein when either GFP-WDR-23 isoform was immunoprecipitated (Fig. 2B), confirming the interaction between WDR-23 and GEN-1. The reciprocal co-IP experiment was performed by immunoprecipitating GEN-1-FLAG, and we observed enrichment of either GFP-WDR-23 isoform, but no enrichment with GFP-(empty vector) (Fig. S2A). This supports the conclusion that WDR-23 and GEN-1 interact biochemically. Furthermore, these data reveal that the N-terminal domain of WDR-23B that mediates nuclear localization does not interfere with binding of GEN-1 *in vitro*.

We wondered whether the interaction between WDR-23 and GEN-1 was conserved in humans. Similar to worms, WDR23 isoforms are spatially distinct, with WDR23-I primarily expressed in the cytoplasm and WDR23-II primarily expressed in the nucleus^17^. After expressing transgenic versions of GFP-WDR23 and FLAG-GEN1 in HEK293T cells, we observed that immunoprecipitating both isoforms of GFP-WDR23 also co-immunoprecipitated FLAG-GEN1 (Fig. 2C), suggesting that the interaction also exists in humans. The reciprocal co-IP experiment was performed by immunoprecipitating FLAG-GEN1, and we observed enrichment of either GFP-WDR23 isoform (Fig. S2B). Importantly, immunoprecipitating GFP-(empty vector) did not enrich for FLAG-GEN1, suggesting that the interaction between WDR23 and GEN1 is specific (Fig. S2C).

The GEN1 structure specific nuclease is one of the four nucleases of the XPG family^33^. Another nuclease in the XPG family is ERCC5, which is involved in the nucleotide excision repair pathway to mend UV-induced damage. Interestingly, a BLAST search revealed that human ERCC5 shares the highest sequence similarity to *C. elegans* GEN-1, and thus, we queried whether WDR23 and ERCC5 interact (Table S2). However, after transfecting GFP-WDR23 and FLAG-ERCC5 in HEK293T cells, immunoprecipitation of GFP-WDR23 did not enrich for FLAG-ERCC5, suggesting no interaction between these two proteins and indicating that the WDR23 interaction with GEN1 is specific (Fig. S2D). Additionally, we were able to co-IP other components of the E3 ubiquitin ligase complex, as IP of either WDR23 isoforms also pulled down DDB1 and CUL4A (Fig. S2E). Based on the biochemical analysis above, we conclude that GEN1 interacts with the WDR23 substrate receptor of the CUL4-DDB1 E3 ubiquitin ligase complex.

### Nuclear WDR-23B, but not cytoplasmic WDR-23A, negatively regulates the DNA repair capacity of GEN-1

Next, we wondered whether the levels of GEN-1 protein would be different in animals with altered expression of the WDR-23 isoforms. We created transgenic worms expressing extra copies of either WDR-23A or WDR-23B in a strain harboring a MosSCI integrated single copy of *gen-1p::gen-1::gfp*. However, after biochemical analysis of protein lysates, we did not see any significant changes with expression of either WDR-23 isoforms (Fig. S3A). We also examined protein lysates of GEN-1::GFP animals after RNAi-depletion of *wdr-23* and did not observe a significant change in steady state level of GEN-1 protein (Fig. S3B), suggesting the possibility of additional layers of GEN-1 regulation beyond WDR-23 in the absence of DNA damage. Additionally, in the study that identified the positive regulation of SLBP *via* WDR23, protein level of SLBP was not affected in WDR23-depleted HeLa cells^20^. This adds further support to the model that WDR23 serves roles beyond protein turnover.

**Figure 3:**
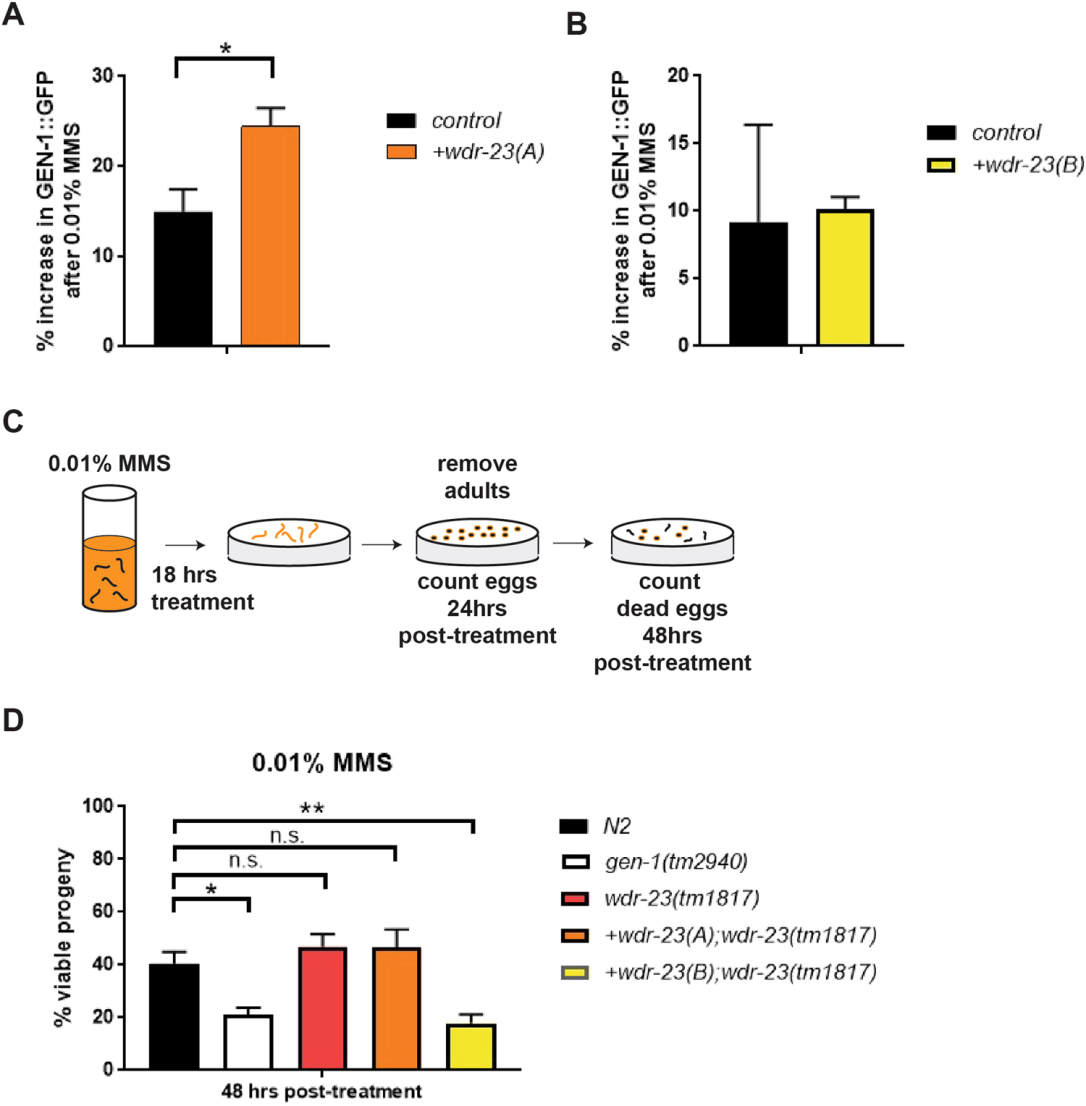
Nuclear WDR-23B negatively impacts GEN-1 DNA repair activity. Quantification of the change in GEN-1 protein abundance in response to DNA damage in animals expressing WDR-23A (**A**; Fig. S3C) or WDR-23B (**B**; Fig. S3D). Western blot images were quantified using ImageJ after normalization to the actin loading control. n = 3 independent biological replicates for each condition. (**C**) Experimental outline to measure DNA repair capacity after treatment with 0.01% MMS to induce DNA damage. (**D**) Animals expressing WDR-23B have decreased viable progeny after DNA damage, which phenocopies *gen-1(tm2940)* DNA repair mutant animals. n ≥ 8 plates of ≥50 animals for each strain. Data are mean ± s.e.m.; one-way ANOVA with multiple comparisons. *p < 0.05, **p < 0.01. (See Table S1 for more details).

To discover whether GEN-1 protein levels differentially change in WDR-23 isoform-specific rescue worms in response to DNA damage, we monitored worms before and after exposure to MMS (Figs 3A,B; S3C,D). MMS is an alkylating agent that creates DNA damage through replication fork collapse^30,34,35^. After biochemical analysis of protein lysates, we observed a slight increase (10–15%) in GEN-1 levels in wildtype worms after DNA damage when compared to untreated worms, suggesting an activating response to the threat of DNA damage (Figs 3A,B; S3C,D). In animals expressing WDR-23B, we observed a less robust percent increase in GEN-1 levels (~10% increase) as compared to wildtype animals after exposure to DNA damage (Figs 3B; S3D). Interestingly, we observed a significantly larger percent increase in GEN-1 protein levels (~24%) in animals with additional WDR-23A expression in response to DNA damage (Figs 3A; S3C). As such, we propose that the expression of cytoplasmic WDR-23A has a positive effect on the response of GEN-1 to DNA damage.

Based on the changes in GEN-1 protein level observed from whole-animal lysates, we wondered whether expression of either isoform of WDR-23 affected the activity of GEN-1. To test the DNA repair capacity of animals with isoform-specific WDR-23 expression, we challenged animals with MMS and measured sterility as a surrogate of failed DNA repair in the germline^30^ (Fig. 3C,D). As previously shown, the *gen-1(tm2940)* null mutants are hypersensitive to DNA damage (Fig. 3D)^30^. We reasoned that if GEN-1 is regulated by WDR-23 isoforms, ablation of both isoforms might lead to MMS hypersensitivity. We found that neither the *wdr-23(tm1817)* null mutant nor the strain solely expressing the WDR-23A isoform are hypersensitive to MMS (Fig. 3D); however, this contrasted with WDR-23B-restricted expression, which did display MMS hypersensitivity (Fig. 3D), a result that hints that the nuclear WDR-23B isoform might regulate GEN-1 by facilitating its turnover, akin to SKN-1 regulation by WDR-23B.

### WDR23 isoforms differentially ubiquitinate GEN1

**Figure 4:**
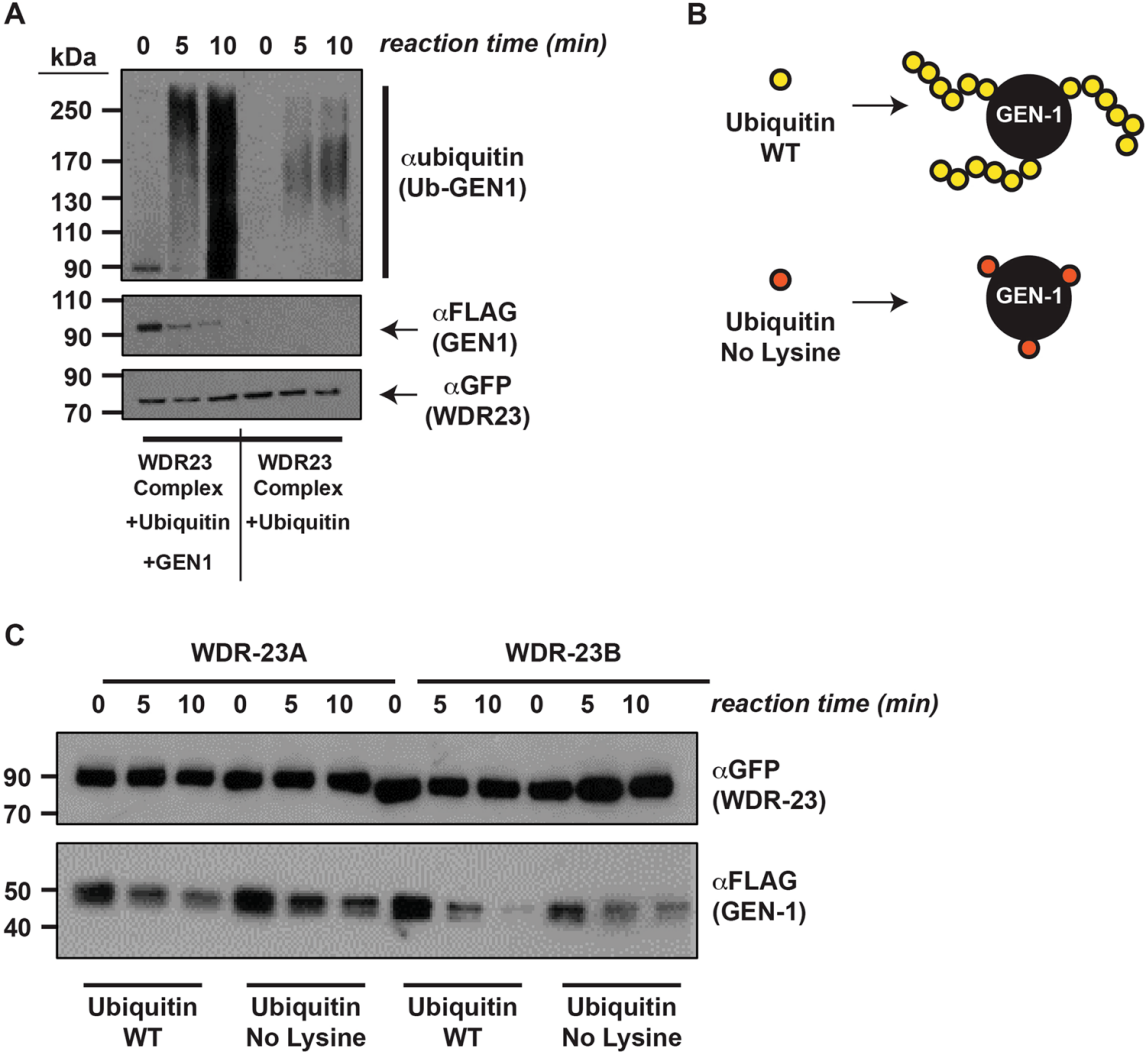
(WDR23) isoforms differentially ubiquitinate (GEN1). (**A**) In the presence of the necessary components for a successful ubiquitination reaction (CUL4A-DDB1-WDR23 complex, UBE1, UBE2D1, ubiquitin, and ATP) GEN1 receives the addition of ubiquitin in a time dependent manner. In the absence of GEN1, less ubiquitination is observed, suggesting minor auto-ubiquitination of the CUL4A-DDB1-WDR23 complex or another unidentified substrate being modified. With the addition of FLAG-GEN1, an increase in the “smear” of ubiquitin is observed over time. Immunoprecipitated samples of GFP-WDR23 isoforms and FLAG-GEN1 were incubated in ubiquitin reaction mixtures and samples were collected at the given time points (0-, 5-, 10-minutes). (**B**) Modified ubiquitin mutant with all lysine mutated to arginine to prevent the formation of chains. (**C**) WDR-23A and WDR-23B differentially modify GEN-1 *in vitro*. Immunoprecipitated samples of GFP-WDR-23 isoforms and GEN-1-FLAG were incubated in ubiquitin reaction mixtures and samples were collected at the given time points (0-, 5-, 10-minutes). αGFP probed for GFP-WDR-23/GFP-WDR23 isoforms. αFLAG for GEN-1-FLAG/FLAG-GEN1. αUbiquitin for ubiquitinated-GEN1/GEN-1.

The number of ubiquitin moieties added onto a substrate can affect its fate; poly-ubiquitination serves as the traditional signal for degradation *via* the proteasome, while mono- and/or multi-mono-ubiquitination can have proteasome-independent outcomes^36,37^. We wondered whether the nuclear and cytoplasmic localized species of human WDR23 would add ubiquitin to human GEN1 in an equivalent manner. As such, we immunoprecipitated both isoforms of GFP-WDR23 in complex with CUL4A and DDB1 (Fig. S2E) and incubated these samples in a reaction mixture containing affinity purified FLAG-GEN1 and the necessary components for efficient ubiquitination^17,20^. After obtaining samples from 0-, 5-, and 10-minute reaction time points, we observed that both isoforms of WDR23 are competent for ubiquitination of GEN1 as a substrate, as indicated by an observed increase in the of ubiquitination signal or “smear” with the addition of FLAG-GEN1 substrate and a corresponding depletion of unmodified GEN1 over the reaction time course (10-minutes) (Figs 4A; S4A). Additional unidentified substrates could co-purify with the IP of GFP-WDR23 in complex with CUL4A and DDB1, as evidenced by the polyubiquitin signal observed in the reaction mix when the GEN1 substrate is excluded (Fig. 4A). However, the increase in polyubiquitin signal observed with the addition of the GEN1 substrate suggests WDR23 facilitates the addition of ubiquitin to GEN1. We noticed that the distribution of ubiquitinated substrates was different between the two isoforms (Fig. S4A), suggestive of a change in pattern of ubiquitin conjugates. Thus, we modified this assay by capitalizing on an ubiquitin mutant where all lysines were mutagenized to arginines (R&D Systems), preventing poly-linkage of ubiquitin, but still allowing for mono-ubiquitination (Fig. 4B). With this paradigm, the ubiquitination pattern of GEN1 was markedly different between the two isoforms of WDR23 (Fig. S4A). We noted a shift in the number of mono-ubiquitin moieties onto GEN1 depending on which of the WDR23 isoforms was utilized; specifically, the cytoplasmic WDR23-I facilitated the addition of more mono-ubiquitin modifications to GEN1 when compared to nuclear WDR23-II, as evidenced by the higher molecular weight species in the presence of WDR23-I (Fig. S4A). These data suggest that the cytoplasmic WDR23-I-CUL4-DDB1 complex could have a preference for adding mono-ubiquitin onto multiple lysines in GEN1.

As an alternative method to quantify the ability of each isoform of WDR-23 to ubiquitinate a substrate, we measured the change in unmodified GEN-1-FLAG over the reaction time course (10-minutes). We applied the same ubiquitin assay, but instead used immunoprecipitation samples from GEN-1-FLAG and GFP-WDR-23 isoforms in the incubation ubiquitin reaction mixture. We observed that both isoforms could modify GEN-1 substrate in the presence of the wild type ubiquitin since unmodified GEN-1 decreases over time (Figs 4C, S4B). With the addition of the ubiquitin mutant, WDR-23A could continue to readily modify GEN-1 throughout the time course (0- to 10-minute time points), as evidenced by the decrease in unmodified GEN-1 (Figs 4C; S4B). In contrast, with the addition of the ubiquitin mutant, WDR-23B could not modify GEN-1 over the same time period since unmodified GEN-1 remains unchanged over time (Figs 4C; S4B). These data highlight the possible function of cytoplasmic WDR-23A to multi-mono-ubiquitinate substrates, while suggesting a reduced ability of nuclear WDR-23B to attach ubiquitin moieties onto multiple sites on GEN-1. Future understanding of the differential mechanisms employed by WDR-23A and WDR-23B for decorating substrates will be of great interest.

## Discussion

CRL complexes are rapidly becoming an attractive option in the treatment of patients with cancer and other age-related diseases^4,7,8,38,39^. However, there is a discrepancy between the number of complexes and their specific ubiquitination substrate(s). Here, we expand the list of known CRL substrates involved in genome stability by identifying a protein critical for DNA repair success as a regulatory target for the CRL substrate receptor WDR23. Specifically, WDR23 was found to interact with and ubiquitinate GEN1, and this interaction was conserved between *C. elegans* and human proteins. The activity and localization of GEN1 must be closely monitored, as unregulated nuclear GEN1 can cause chromosomal abnormalities^31,40,41^, and loss of GEN1 has been observed in ovarian and colon cancer cell lines^42^. We suggest that WDR23 participates in the regulation of GEN1 to avoid these dysfunctional processes, thus allowing cells to maintain a proper response system to DNA damage.

Previously, WDR23 was identified as a key component during DNA replication by enhancing histone biogenesis through controlling the activity of its substrate, called stem-loop binding protein (SLBP)^20^. Mutating multiple lysines on SLBP impaired histone levels, while single ubiquitin attachments were detected in an ubiquitin assay in combination with WDR23, positing mono-ubiquitination rather than poly-ubiquitination *via* WDR23. This could be attributed to the primary function of cytoplasmic WDR23-I as a positive regulator of its substrates. On the other hand, depletion of WDR23 led to the accumulation of p21^19^, while WDR23 overexpression decreased NRF2 protein levels^17^, suggesting a role for WDR23 in regulating protein stability. We attribute this phenomenon to nuclear WDR23-II activity in ensuring efficient protein turnover.

The isoforms of WDR-23 share similar sequences that vary only in the N-terminal region. Interestingly, cytoplasmic WDR-23A is predicted to encode two nuclear export signals (NES) in the N-terminal region that are not found in nuclear WDR-23B. This hypothetical NES in cytoplasmic WDR-23A could help explain its localization primarily in the cytoplasm, but future studies are needed to validate this claim. Furthermore, identifying any nuclear localization signal(s) present in nuclear WDR-23B would be advantageous in clarifying the model describing the differences in protein regulation between isoforms of WDR-23.

**Figure 5:**
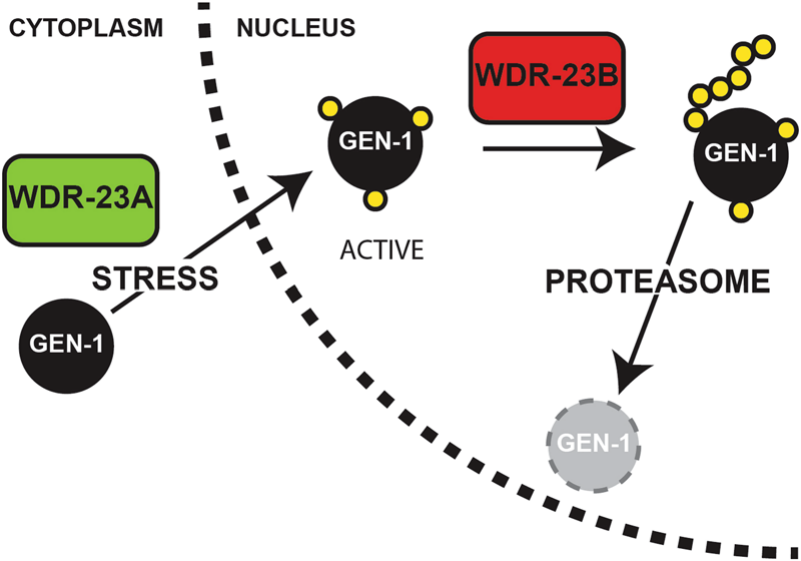
WDR-23 isoforms regulate GEN-1 and/or SKN-1 stability and activity. Model for the coordination between the nuclear and cytoplasmic isoforms of WDR-23-mediated regulation of its substrates. Cytoplasmic WDR-23A activity results in activation of substrates, while nuclear WDR-23B drives turnover of substrates to the proteasome. The activity of cytoplasmic (activating) and nuclear (repressive) WDR-23 on its substrates may be linked to mono- or poly-ubiquitination, respectively.

Our data supports a spatially-dependent model of regulation where cytoplasmic or nuclear WDR-23 differentially affects substrates. The interplay between these two WDR-23 isoforms will require further study, as the data suggest that cytoplasmic WDR-23A activity may occur before nuclear WDR-23B activity. Our proposed model suggests that cytoplasmic WDR23 can prime or activate substrate(s) by adding multi-mono-ubiquitin onto several lysines. Through an unknown and possibly unrelated mechanism of nuclear localization, these activated substrates become targets of nuclear WDR-23 for proteasomal degradation *via* poly-ubiquitin linkage (Fig. 5). Our data provide an explanation to the multi-functional role of WDR23. However, we cannot rule out the possibility that either isoform is capable of serving both functions, dependent on the cellular state.

Additionally, the variation in activation of SKN-1 target genes *gst-4* and *gcs-1* observed in the *wdr-23(tm1817)* null mutant could be attributed to the dynamics and localization of the isoforms of WDR-23 present in different tissues. We lose this valuable information from whole-animal lysis, and thus the functional differences in each isoform of WDR-23 between tissues is still unknown. Future studies could examine SKN-1 activity *via* target gene expression with restoration of either isoform of WDR-23 in specific tissues, such as muscle, intestine, or hypodermis. Furthermore, a previous study observed WDR-23A expression localized to mitochondria^16^, suggesting functional differences between isoforms of WDR-23.

This is not the first discovery of an E3 ubiquitin ligase complex having both proteasome and proteasome-independent functions^43–45^. A CRL complex was observed to negatively regulate the oncogenic transcription factor FOXM1, while the substrate receptor (DCAF1) was shown to be required for FOXM1 activation^43^. Additionally, another E3 ubiquitin ligase component (SKP2) was shown to regulate both the stability and activity of its substrate (c-MYC)^44,45^. However, a substrate’s fate has not been shown to be dependent on the spatial localization of the CRL complex, as DCAF1 and SKP2 are not strictly confined to either the cytoplasm or nuclear compartments. We believe a “cross-talk” exists between WDR23 isoforms that facilitates the regulation of its substrates in times of cellular stress (i.e. DNA damage and/or oxidative stress).

Maintaining protein regulation in response to oxidative stress and DNA damage inducing agents is critical to ensure a healthy cellular environment. Our findings highlight the functional differences between the two isoforms of WDR23 in order to maintain efficient and accurate regulation of both the oxidative stress and DNA repair pathways. The discovery of additional ubiquitination substrates for nuclear and cytoplasmic forms of WDR23 will be of great interest.

## Materials and Methods

### *C. elegans* strains and maintenance

*C. elegans* were maintained using standard techniques^46^. Strains used include: wildtype *N2* Bristol, CL2166*(gst-4p::gfp*), *wdr-23(tm1817) I*, *gen-1(tm2940) III*, OJ1507: vjEx594 genomic *wdr-23*, OJ1678*: wdr-23(tm1817 I); wdr-23p::wdr-23A II*^16^, OJ1679*: wdr-23(tm1817) I; wdr-23p::wdr-23B II*^16^, TG2511*: gen-1(tm2940) III*, *gtSi02[Pgen-1::GEN-1::GFP::gen-1*; *cb-unc-119(* + *)] II; unc-119(ed3) III*. Double mutants were generated by standard genetic techniques. OJ1678 was crossed to OJ1679 to generate trans-heterozygous *wdr-23(tm1817) I;[wdr-23p::wdr-23A II]/[wdr-23p::wdr-23B] II* animals for reproduction assays. Similarly, OJ1678 or OJ1679 was crossed to TG2511 to generate overexpression of WDR-23 isoforms with the *gen-1p::gen-1::gfp* reporter.

### Reproduction assays

L4 stage animals of each strain were moved to their own respective experimental plate, and their reproductive output was tracked, twice daily, by moving each animal to a fresh plate every twelve hours until reproduction ceased. To ensure accurate counts of progeny number, each plate was assessed at least twice, 24- to 48-hrs after the hermaphrodite mother was moved from the plate.

### Yeast two-hybrid screen

Yeast two-hybrid screen was performed according to the manufacturer’s protocol (Clonetech). Y2H gold cells were combined with bait plasmid DNA (*C. elegans wdr-23A* cDNA in pLexA) and prey plasmid DNA (*C. elegans* cDNA library in pACT2.2). ~200,000 colonies were screened for both X-α-galactosidase activity and Aureobasidin A resistance. Positive clones were retested with the additional requirement of growth in the absence of histidine. Plasmids were isolated for positive colonies and sequenced to identify potential interactors using primers supplied by the vendor that anneal to the multiple cloning site where cDNAs were inserted. Sequenced confirm clones were retested for interaction with the *wdr-23A* bait plasmid.

### Fluorescence assays

L4-staged or later animals expressing *gst-4p::gfp* were placed on microscopic slides and imaged using a Zeiss Axio Imager.M2m microscope, Axio Cam MRm camera, and Zen Blue software. All images were taken with a GFP-filter set to 20 ms exposures.

Fluorescent measurements were made using ImageJ and normalized to body size. The “straighten” and “collage” functions in ImageJ were used for presentation.

### DNA damage and oxidative stress assays

For DNA damage assessment, L4 worms were incubated with 0.01% MMS in M9 for 18-hrs. 24-hrs later, adults were placed on new agar plates and allowed to lay eggs overnight. Then, adults were removed and progeny were counted. 24-hrs later, the number of viable progeny were counted and viable progeny percentages were calculated.

For oxidative stress assessment, L4 worms were incubated with 10 mM H2O2 in M9 for 30 mins. 24-hrs later, the number of living adults were counted and survival percentages were calculated.

### RNAi treatment

NGM plates containing 5 mM IPTG and 100 µg/ml carbenicillin were seeded with cultures from double-stranded RNAi-expressing HT115 bacteria.

### RNA extraction and quantitative PCR

Quantitative PCR was performed as previously described^47^. RNA was extracted according to the manufacturer’s protocol (Zymo Research). RNA was reverse-transcribed to complementary DNA using qScript cDNA Supermix (Quanta Biosciences).

**Table:** 

**gene**	**Fwd primer (5′-3′)**	**Rev primer (5′-3′)**
*snb-1*	CCGGATAAGACCATCTTGACG	GACGACTTCATCAACCTGAGC
*gst-4*	GCTGAGCCAATCCGTATCAT	CCGAATTGTTCTCCATCGAC
*gcs-1*	CCAATCGATTCCTTTGGAGA	TCGACAATGTTGAAGCAAGC

### Cell culture maintenance and techniques

Cell cultures were maintained as previously described^48^. HEK-293T cells were cultured at 37 degrees Celsius (5% CO2) in Dulbecco’s modified Eagle’s medium supplemented with 10% fetal bovine serum and 1% antibiotic/antimycotic (Thermo Fisher). Transfections were performed according to the manufacturer’s protocol with Lipofectamine 3000 (Thermo Fisher). Full-length cDNA sequence of human WDR23 isoforms I and II, and *C. elegans* WDR-23 isoforms A and B were cloned into pcDNA 6.2/N-EmGFP/TOPO (Thermo Fisher). Human 3XFLAG:GEN1 and *C. elegans* GEN-1-3XFLAG were purchased from GeneCopeia.

### Co-immunoprecipitation

Co-IP *via* GFP trap beads (Chromotek) were performed as previously described^17^. Additionally, co-IP *via* FLAG-M2 affinity resin (Sigma) were completed according to the manufacturer’s protocol (Sigma). Briefly, HEK-293T cells were lysed in 0.5% CHAPS buffer (10 mM Tris/Cl pH 7.5, 150 mM NaCl, 0.5 mM EDTA, 0.5% CHAPS) containing Halt protease inhibitor (Thermo Fisher). A small aliquot of cell lysates were saved for “input whole-cell extract” analysis later – 2.5% of the total lysate was loaded as input to assess enrichment following IP. Cell lysates were incubated with magnetic FLAG beads for 2 hours at 4 degrees Celsius. Before the washing step, a small aliquot of unbound/flow through cell lysate were saved for later analysis. After three washes, immunoprecipitated protein complexes were eluted with 2X sample buffer (0.1 M Tris/Cl pH 6.8, 4% SDS, 20% glycerol, 0.2 M DTT, 0.1% bromophenol blue) by boiling for 5 minutes at 95 degrees Celsius. Samples were analyzed by Western blot.

### Western blot analysis and antibodies

For gel electrophoresis studies, fifty L4 stage animals were picked and lysed in protein lysis buffer (Thermo Fisher) at 95 degrees Celsius for 10 min followed by centrifugation at 12,000 × g for 10 min to remove insoluble material. Cell lysates were run on Bolt 4–12% bis-tris polyacrylamide gels in MOPS running buffer (Thermo Fisher) and transferred to nitrocellulose membranes. Membranes were blocked in 5% milk in 1XPBST for 1 hour at room temperature. Antibodies were blotted overnight at 4 degrees Celsius and include: GFP GF28R and Ubiquitin 1859660 (Thermo Fisher); FLAG M2 (Sigma); CUL4A 113876 (GeneTex), DDB1 A300–462 (Bethyl), and Actin A5441 (Sigma).

Western blot images from Supplemental Fig. 3 were quantified using ImageJ software with antibodies against actin as the loading control. Briefly, regions of interest were created individually for GFP protein bands (*gen-1::gfp*) and for actin (loading control). Pixel density were recorded using the “*command* + *m*” function in ImageJ and data were exported and analyzed in Microsoft Excel. In Fig. S4B, a similar method was used for the quantification of un-modified GEN-1 seen in Fig. 4C, but instead we quantified proteins bands from GEN-1-FLAG (via antibodies against FLAG) and used protein bands from GFP-WDR-23 isoforms as the loading control (*via* antibodies against GFP).

Digital images of western blots are from single gel analyses, were not digitally adjusted, and were only cropped around the edges to remove empty areas of the image. Refer to Supplementary Data 1 for unmodified original images.

### *In vitro* ubiquitination assay

*In vitro* ubiquitination reactions were done as previously described^20^. Briefly, FLAG-tagged GEN1(GEN-1) or GFP-tagged WDR23(WDR-23) isoforms were immunoprecipitated from HEK-293T cells as described above, with the exception that GEN1 (GEN-1) was eluted by competition with FLAG peptide (Sigma). Purified components were incubated with UbE1, E2, and ubiquitin^WT^ or ubiquitin^noK^ (R&D Systems) and incubated in reaction buffer (50 mM Tris pH 7.6, 3 mM ATP, 0.5 mM DTT, 10 mM MgCl2, and 1 mg/ml BSA) at 37 degrees Celsius for 10 minutes total. 15 ul of the reaction mixture was removed at each time point (0-, 5-, and 10-minutes), resolved by gel electrophoresis and proteins detected by immunoblotting with the indicated monospecific antibodies (described above).

### Statistics

Data were analyzed using one-way ANOVA and unpaired Student’s t-test (p < 0.05) in GraphPad Prism 7 software.

## Supplementary information

Table S1

Dataset 1

Supplementary Figures

## References

[1] Mészáros B, Kumar M, Gibson TJ, Uyar B, Dosztányi Z (2017). Degrons in cancer. Sci. Signal.

[2] Labbadia J, Morimoto RI (2015). The Biology of proteostasis in aging and disease. Annu. Rev. Biochem..

[3] Song L, Luo Z (2019). Post-translational regulation of ubiquitin signaling. J. Cell Biol..

[4] Zheng N, Shabek N (2017). Ubiquitin Ligases: structure, function, and regulation. Annu. Rev. Biochem..

[5] Li W (2008). Genome-wide and functional annotation of human E3 ubiquitin ligases identifies MULAN, a mitochondrial E3 that regulates the organelle’s dynamics and signaling. PLoS One..

[6] Zimmerman ES, Schulman BA, Zheng N (2010). Structural assembly of cullin-RING ubiquitin ligase complexes. Curr. Opin. Struct. Biol..

[7] Lee J, Zhou P (2007). DCAFs, the missing link of the CUL4-DDB1 ubiquitin ligase. Mol. Cell.

[8] Zhao Y, Sun Y (2013). Cullin-RING ligases as attractive anti-cancer targets. Curr. Pharm. Des..

[9] Havens CG, Walter JC (2011). Mechanism of CRL4 Cdt2, a PCNA-dependent E3 ubiquitin ligase. Genes Dev..

[10] Groisman R (2003). The ubiquitin ligase activity in the DDB2 and CSA complexes is differentially regulated by the COP9 signalosome in response to DNA damage. Cell.

[11] Sugasawa K (2005). UV-induced ubiquitylation of XPC protein mediated by UV-DDB-ubiquitin ligase complex. Cell.

[12] Kim Y, Starostina NG, Kipreos ET (2008). The CRL4 Cdt2 ubiquitin ligase targets the degradation of p21 Cip1 to control replication licensing. Genes Dev..

[13] Nag A, Bagchi S, Raychaudhuri P (2004). Cul4A physically associates with MDM2 and participates in the proteolysis of p53. Cancer Res..

[14] Wang H (2006). Histone H3 and H4 ubiquitylation by the CUL4-DDB-ROC1 ubiquitin ligase facilitates cellular response to DNA damage. Mol. Cell.

[15] Petroski MD, Deshaies RJ (2005). Function and regulation of cullin-RING ubiquitin ligases. Nat. Rev. Mol. Cell Biol..

[16] Staab Trisha A., Griffen Trevor C., Corcoran Connor, Evgrafov Oleg, Knowles James A., Sieburth Derek (2013). The Conserved SKN-1/Nrf2 Stress Response Pathway Regulates Synaptic Function in Caenorhabditis elegans. PLoS Genetics.

[17] Lo JY, Spatola BN, Curran SP (2017). WDR23 regulates NRF2 independently of KEAP1. PLoS Genet..

[18] Hasegawa, K. & Miwa, J. Genetic and cellular characterization of Caenorhabditis elegans mutants abnormal in the regulation of many phase II enzymes. *PLoS One* **5** (2010). 10.1371/journal.pone.0011194 PMC2887452 20585349

[19] Chen Z (2017). CRL4BDCAF11E3 ligase targets p21 for degradation to control cell cycle progression in human osteosarcoma cells. Sci. Rep..

[20] Brodersen MML (2016). CRL4WDR23-mediated SLBP ubiquitylation ensures histone supply during DNA replication. Mol. Cell.

[21] Choe KP, Przybysz AJ, Strange K (2009). The WD40 repeat protein WDR-23 functions with the CUL4/DDB1 ubiquitin ligase to regulate nuclear abundance and activity of SKN-1 in Caenorhabditis elegans. Mol. Cell Biol..

[22] Sykiotis GP, Bohmann D (2010). Stress-activated cap’ n’ collar transcription factors in aging and human disease. Sci. Signal.

[23] Curran SP, Ruvkun G (2007). Lifespan regulation by evolutionarily conserved genes essential for viability. PLoS Genet..

[24] Tang L, Choe KP (2015). Characterization of skn-1/wdr-23 phenotypes in Caenorhabditis elegans; pleiotrophy, aging, glutathione, and interactions with other longevity pathways. Mech. Ageing Dev..

[25] An JH, Blackwell TK (2003). SKN-1 links C. elegans mesendodermal specification to a conserved oxidative stress response. Genes Dev..

[26] Lynn Dana A., Dalton Hans M., Sowa Jessica N., Wang Meng C., Soukas Alexander A., Curran Sean P. (2015). Omega-3 and -6 fatty acids allocate somatic and germline lipids to ensure fitness during nutrient and oxidative stress inCaenorhabditis elegans. Proceedings of the National Academy of Sciences.

[27] Dalton HM, Curran SP (2018). Hypodermal responses to protein synthesis inhibition induce systemic developmental arrest and AMPK-dependent survival in Caenorhabditis elegans. PLoS Genet..

[28] Paek J (2012). Mitochondrial SKN-1/Nrf mediates a conserved starvation response. Cell Metab..

[29] Ip SCY (2008). Identification of Holliday junction resolvases from humans and yeast. Nature.

[30] Bailly AP (2010). The Caenorhabditis elegans homolog of Gen1/Yen1 resolvases links DNA damage signaling to DNA double-strand break repair. PLoS Genet..

[31] Chan YW, West SC (2014). Spatial control of the GEN1 Holliday junction resolvase ensures genome stability. Nat. Commun..

[32] Blanco MG, Matos J, West SC (2014). Dual control of Yen1 nuclease activity and cellular localization by Cdk and Cdc14 prevents genome instability. Mol. Cell.

[33] Grasby JA, Finger LD, Tsutakawa SE, Atack JM, Tainer JA (2012). Unpairing and gating: sequence-independent substrate recognition by FEN superfamily nucleases. Trends Biochem. Sci..

[34] Craig AL, Moser SC, Bailly AP, Gartner A (2012). Methods for Studying the DNA Damage Response in the Caenorhabditis elegans Germ Line. Methods Cell Biol..

[35] Holway, A. H., Kim, S., Volpe, A. L & Michael, W. M. Checkpoint silencing during the DNA damage response in Caenorhabditis elegans embryos. *J. Cell Biol*. **172** (2006). 10.1083/jcb.200512136 PMC2063758 16549501

[36] Swatek KN, Komander D (2016). Ubiquitin modifications. Cell Res..

[37] Thrower JS, Hoffman L, Rechsteiner M, Pickart CM (2000). Recognition of the polyubiquitin proteolytic signal. EMBO J.

[38] Wu K (2016). Suramin inhibits cullin-RING E3 ubiquitin ligases. PNAS.

[39] Sharma P, Nag A (2014). CUL4A ubiquitin ligase: a promising drug target for cancer and other human diseases. Open Biol..

[40] Sarbajna S, Davies D, West SC (2014). Roles of SLX1-SLX4, MUS81-EME1, and GEN1 in avoiding genome instability and mitotic catastrophe. Genes Dev..

[41] Wechsler T, Newman S, West SC (2011). Aberrant chromosome morphology in human cells defective for Holliday junction resolution. Nature.

[42] Wood L (2007). The Genomic Landscapes of human breast and colorectal cancers. Science.

[43] Wang X (2017). VprBP/DCAF1 regulates the degradation and nonproteolytic activation of the cell cycle transcription factor FoxM1. Mol. Cell Biol..

[44] Von Der Lehr N (2003). The F-Box protein Skp2 participates in c-Myc proteosomal degradation and acts as a cofactor for c-Myc-regulated transcription. Mol. Cell.

[45] Kim SY, Herbst A, Tworkowski KA, Salghetti SE, Tansey WP (2003). Skp2 regulates Myc protein stability and activity. Mol. Cell.

[46] Sulston JE, Brenner S (1974). The DNA of *Caenorhabditis elegans*. Genetics.

[47] Pang S, Curran SP (2014). Adaptive capacity to bacterial diet modulates aging in C. elegans. Cell Metab..

[48] Khanna A, Johnson DL, Curran SP (2014). Physiological roles for mafr-1 in reproduction and lipid homeostasis. Cell Rep..

