# Supplementary material for: Nuclear and cytoplasmic WDR-23 isoforms mediate differential effects on GEN-1 and SKN-1 substrates
Source: Sci Rep. 2019 Aug 13;9:11783. doi: 10.1038/s41598-019-48286-y (PMC6692315; doi:10.1038/s41598-019-48286-y)
Supplement: Supplementary file 2 — Dataset 1 [file 41598_2019_48286_MOESM2_ESM.pdf]

Nuclear and cytoplasmic WDR-23 isoforms mediate differential effects on GEN-1 and SKN-1 substrates

Brett N. Spatola[1,2], Jacqueline Y. Lo[1,2,3], Bin Wang[4], and Sean P. Curran[1,2,5,*]

Supplementary Dataset 1

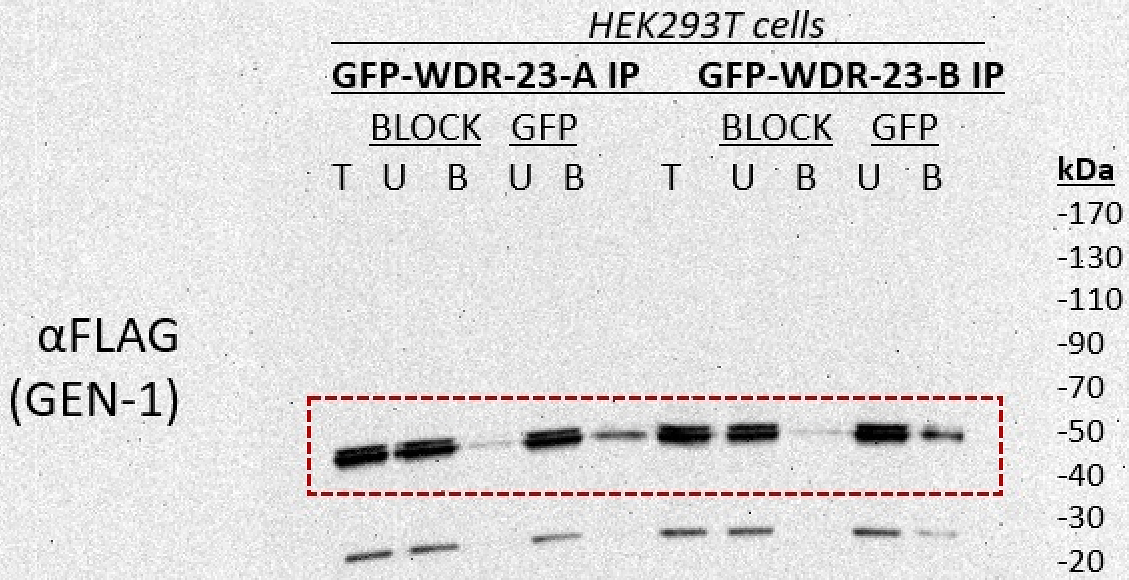
HEK293T cells
GFP-WDR-23-A IP
GFP-WDR-23-B IP
BLOCK
GFP
BLOCK
GFP
T U B U B
T U B U B
kDa
-170
-130
-110
-90
-70
-50
-40
-30
-20
αFLAG
(GEN-1)

fig2b_flag

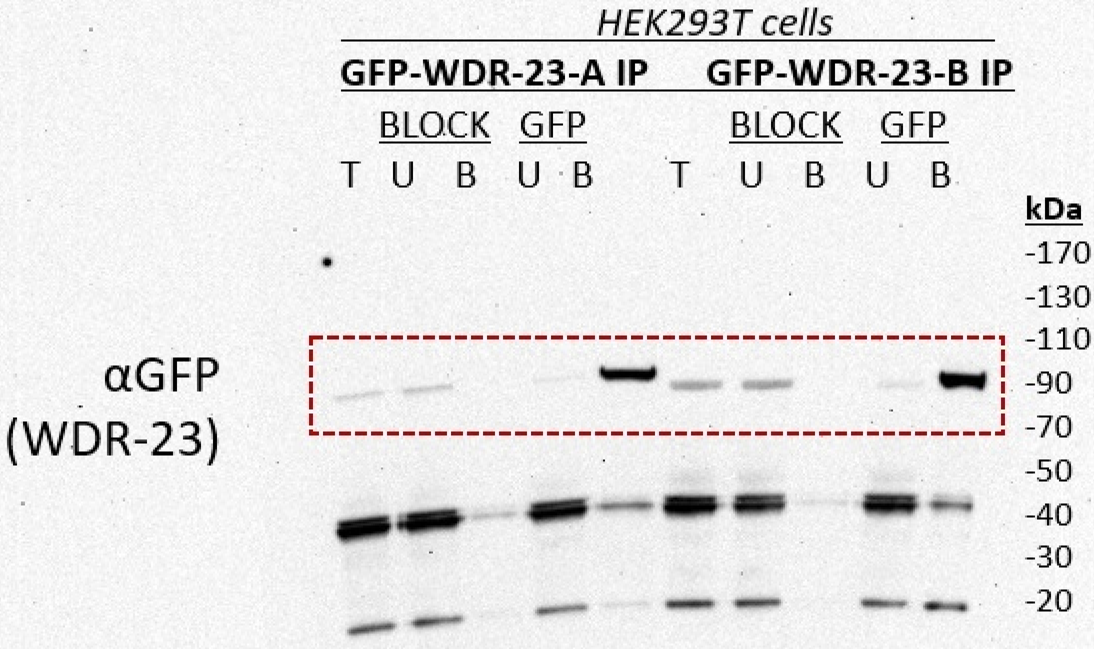
HEK293T cells
GFP-WDR-23-A IP
GFP-WDR-23-B IP
BLOCK
GFP
BLOCK
GFP
T U B U B T U B U B
kDa
-170
-130
-110
-90
-70
-50
-40
-30
-20
αGFP
(WDR-23)

fig2b_gfp

αFLAG
(GEN1)

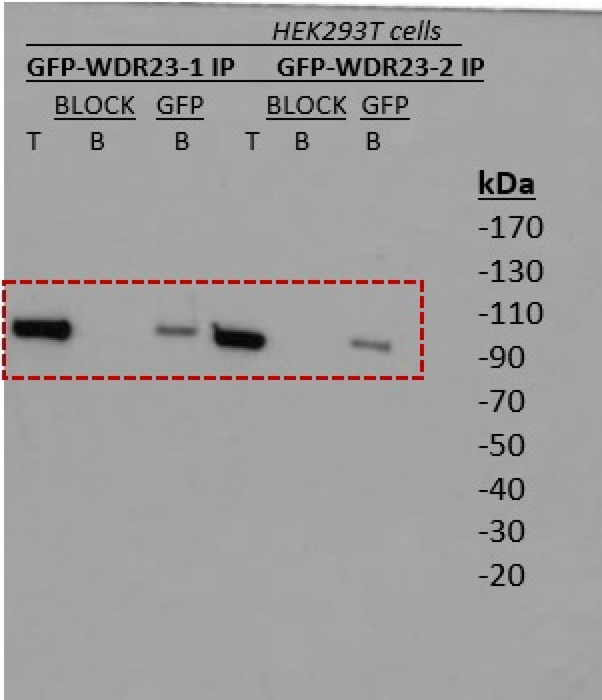

fig2c_flag

αGFP
(WDR23)

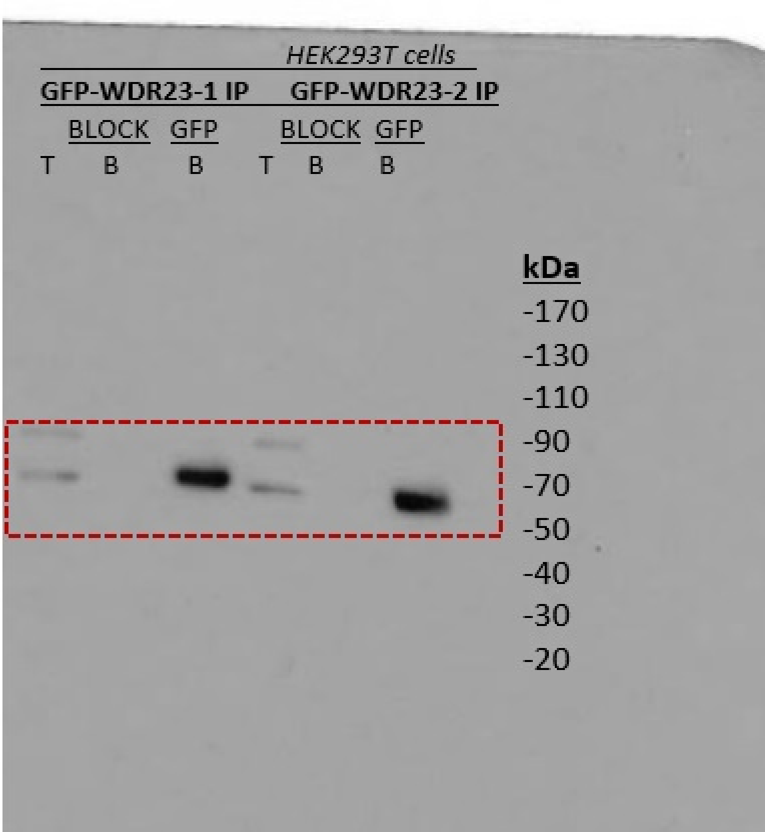

fig2c_gfp

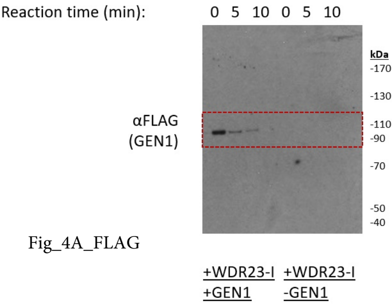
Reaction time (min):
0 5 10 0 5 10
kDa
-170
-130
-110
-90
-70
-50
-40
αFLAG
(GEN1)
+WDR23-I
+GEN1
+WDR23-I
-GEN1

Fig_4A_FLAG

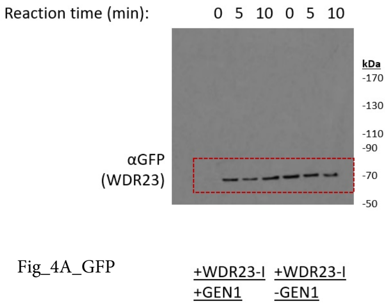
Reaction time (min):
0 5 10 0 5 10
kDa
-170
-130
-110
-90
-70
-50
αGFP
(WDR23)
+WDR23-I
+GEN1
+WDR23-I
-GEN1

Fig_4A_GFP

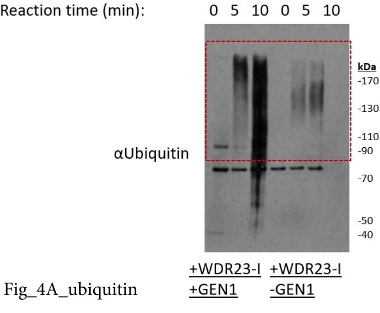

Fig_4A_ubiquitin

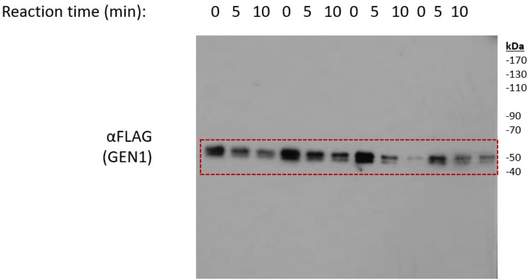

Fig_4C_FLAG

+Ub(wt) +Ub(Lys) +Ub(wt) +Ub(Lys)

+WDR-23A +WDR-23B

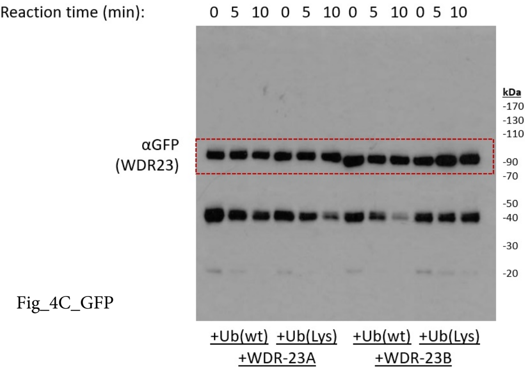
Reaction time (min):
0 5 10 0 5 10 0 5 10 0 5 10
kDa
-170
-130
-110
-90
-70
-50
-40
-30
-20
αGFP
(WDR23)
+Ub(wt) +Ub(Lys)
+WDR-23A
+Ub(wt) +Ub(Lys)
+WDR-23B

Fig_4C_GFP

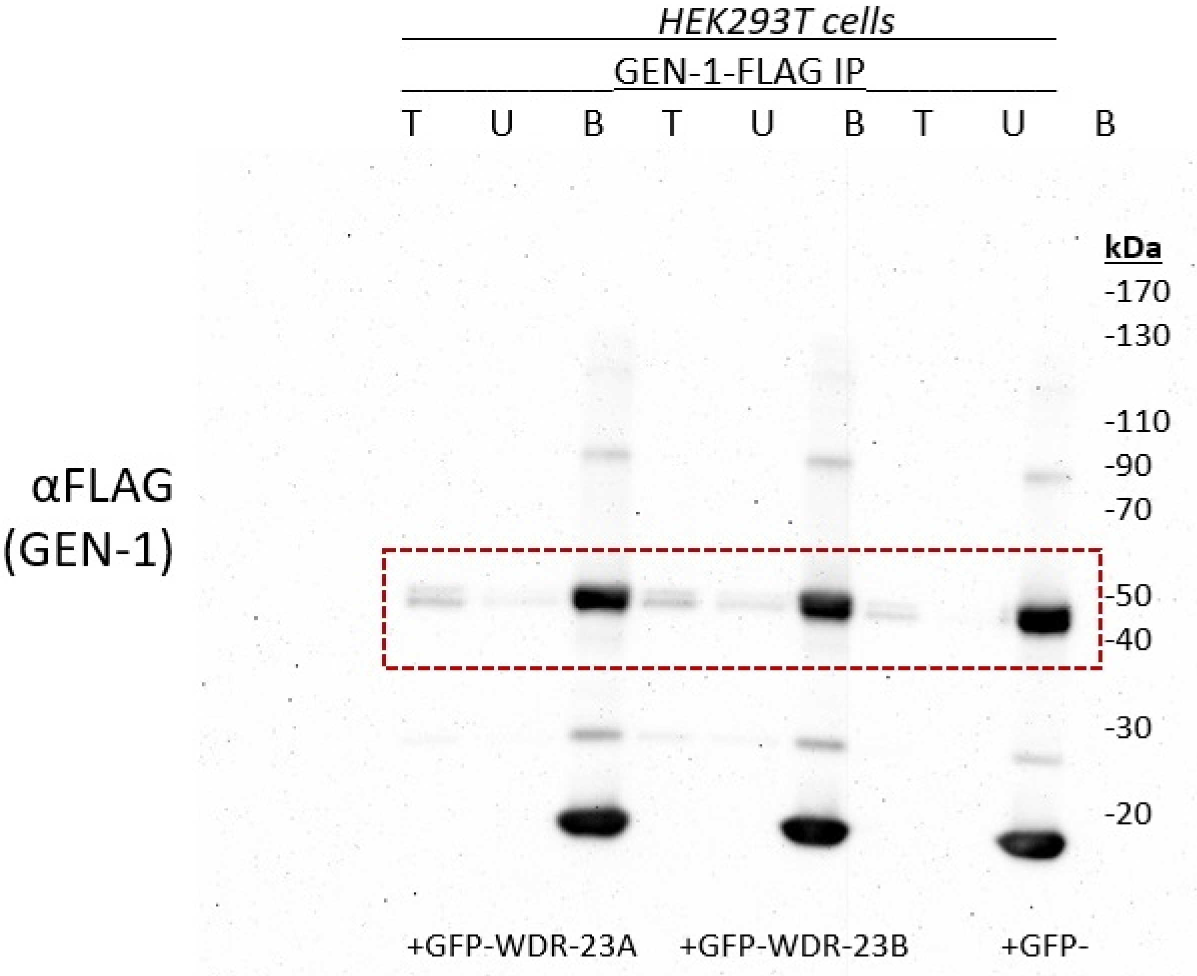
HEK293T cells
GEN-1-FLAG IP
T U B T U B T U B
kDa
-170
-130
-110
-90
-70
-50
-40
-30
-20
αFLAG
(GEN-1)
+GFP-WDR-23A
+GFP-WDR-23B
+GFP-

fig_S2_A_FLAG

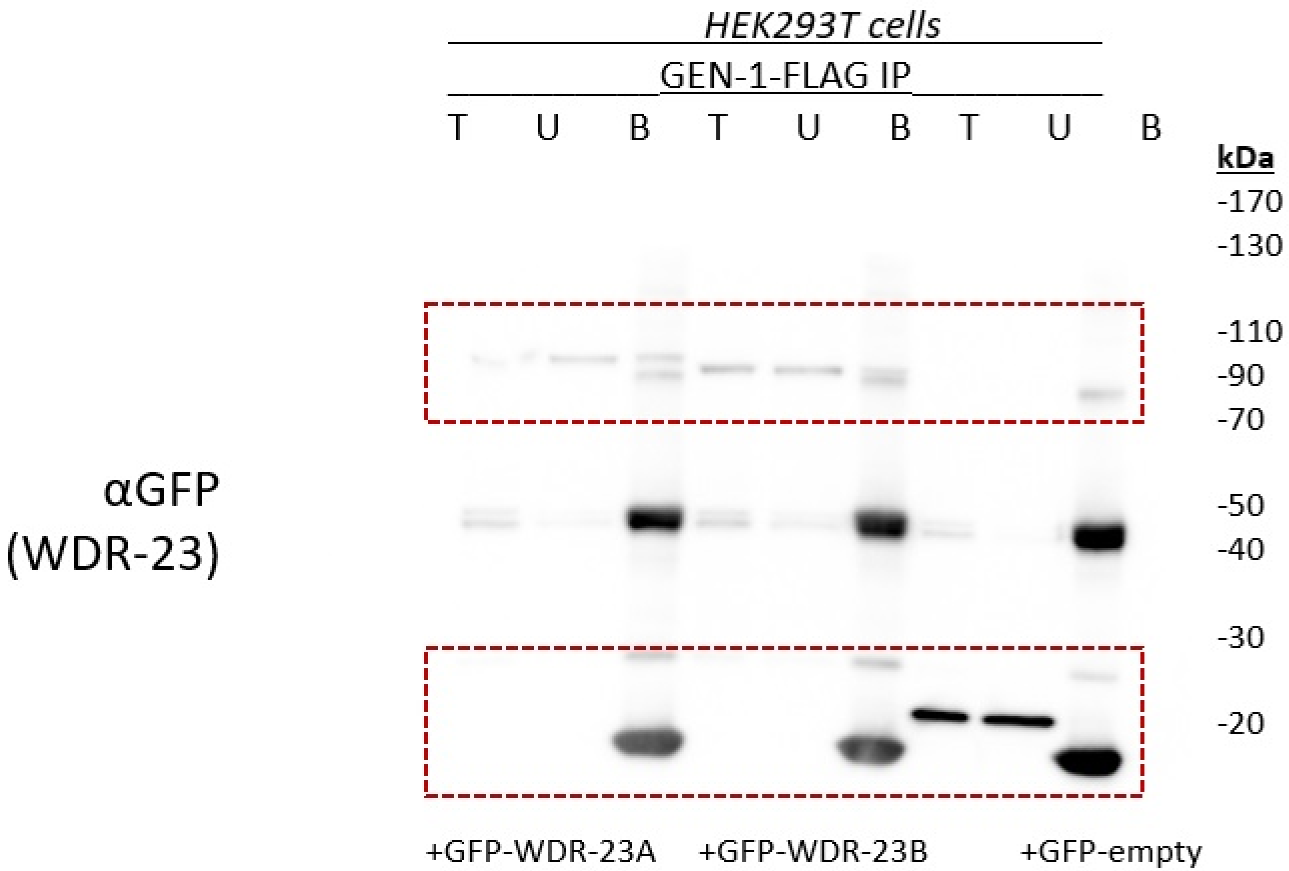

HEK293T cells
GEN-1-FLAG IP
T U B T U B T U B
kDa
-170
-130
-110
-90
-70
-50
-40
-30
-20
αGFP
(WDR-23)
+GFP-WDR-23A
+GFP-WDR-23B
+GFP-empty

fig_S2_A_GFP

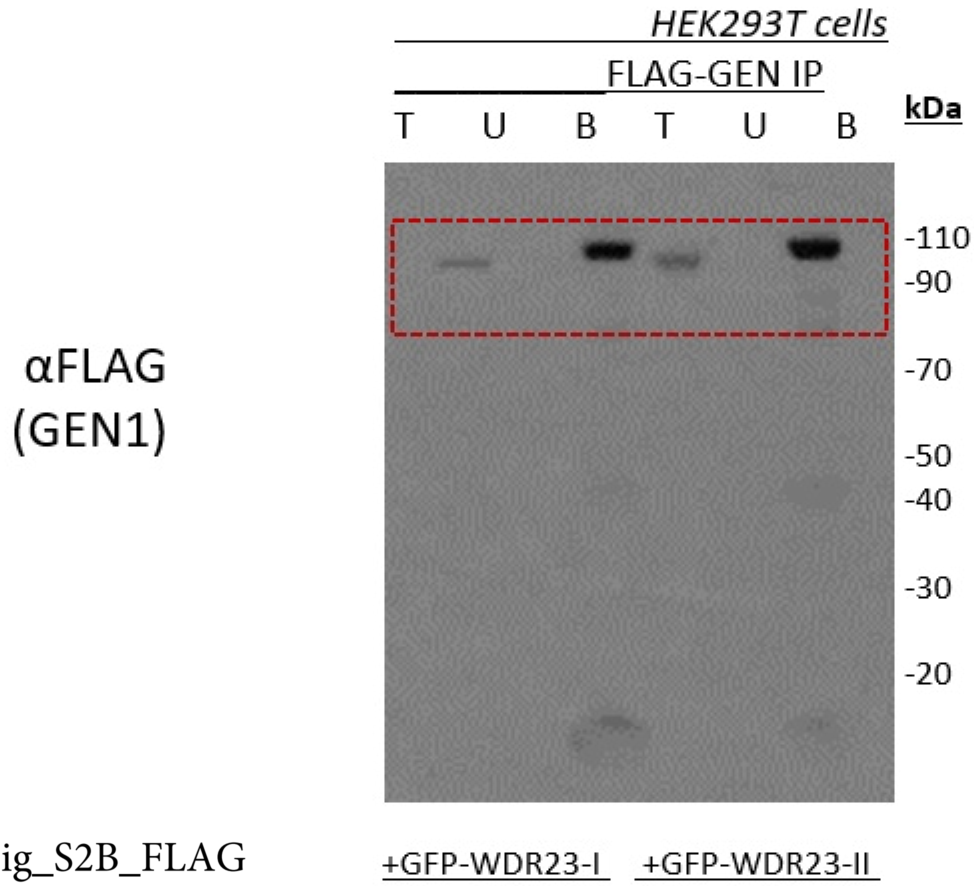

HEK293T cells
FLAG-GEN IP
T U B T U B
kDa
-110
-90
-70
-50
-40
-30
-20
αFLAG
(GEN1)
+GFP-WDR23-I
+GFP-WDR23-II

Fig_S2B_FLAG

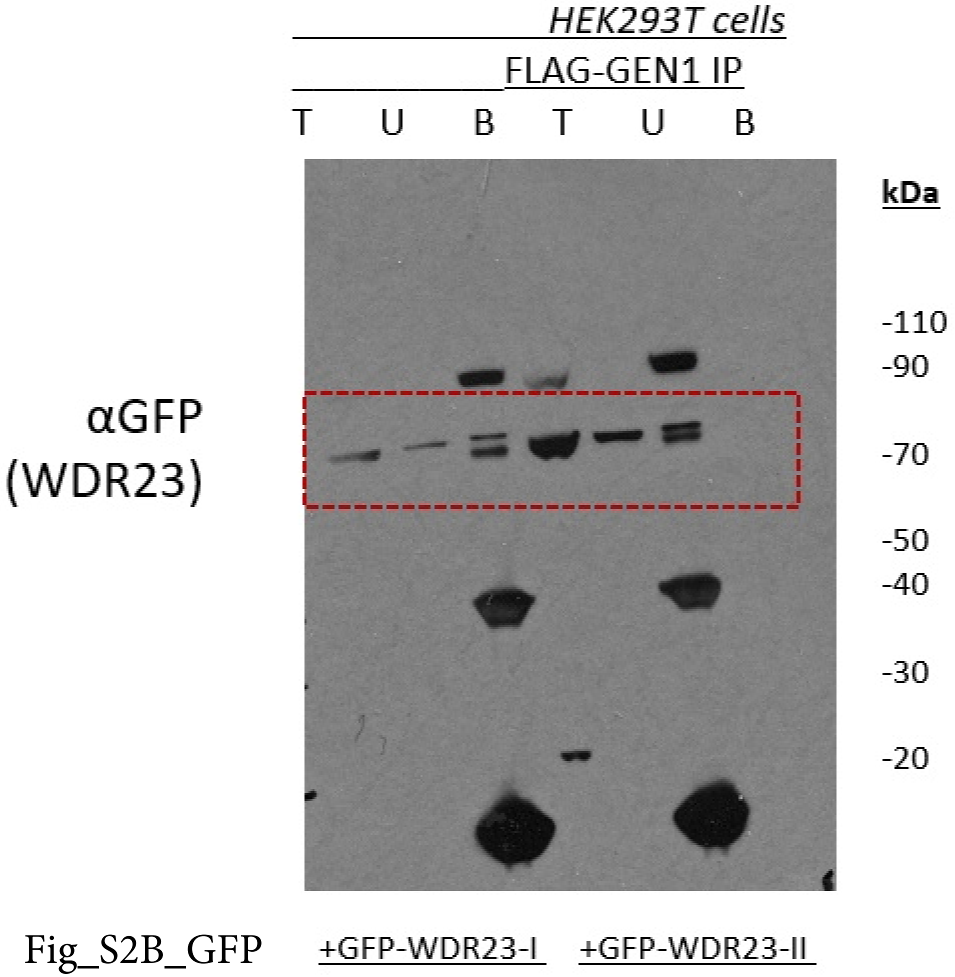
HEK293T cells
FLAG-GEN1 IP
T U B T U B
kDa
-110
-90
-70
-50
-40
-30
-20
αGFP
(WDR23)
+GFP-WDR23-I
+GFP-WDR23-II

Fig_S2B_GFP

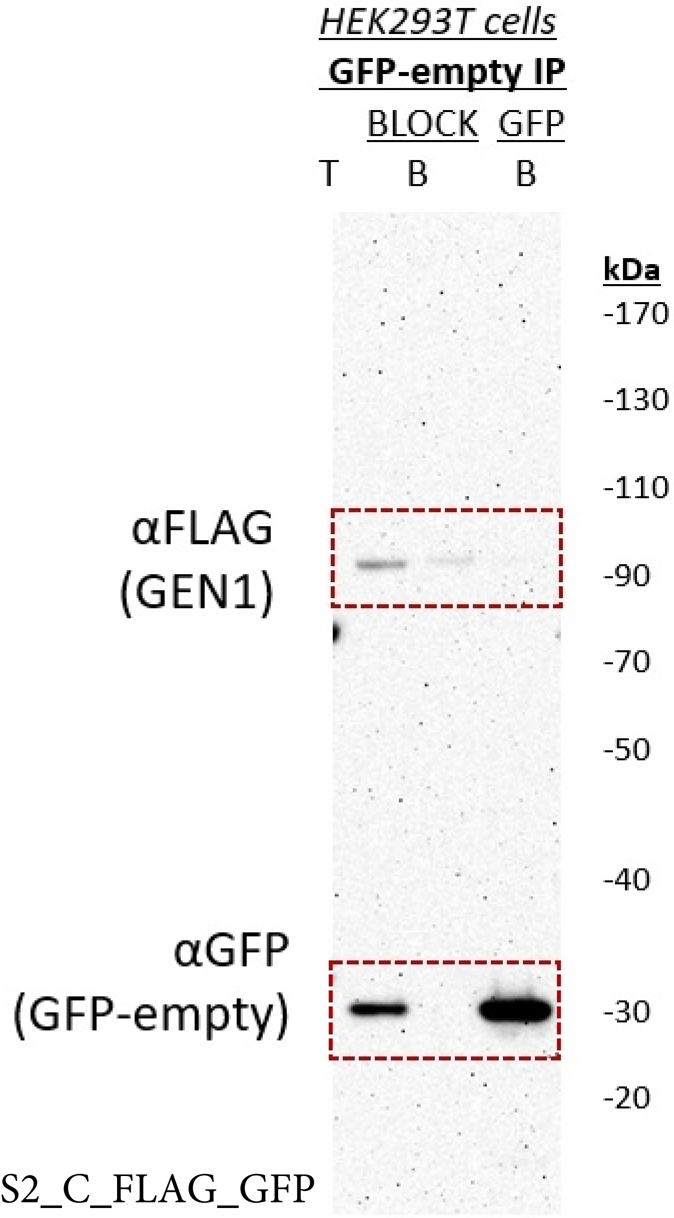

fig_S2_C_FLAG_GFP

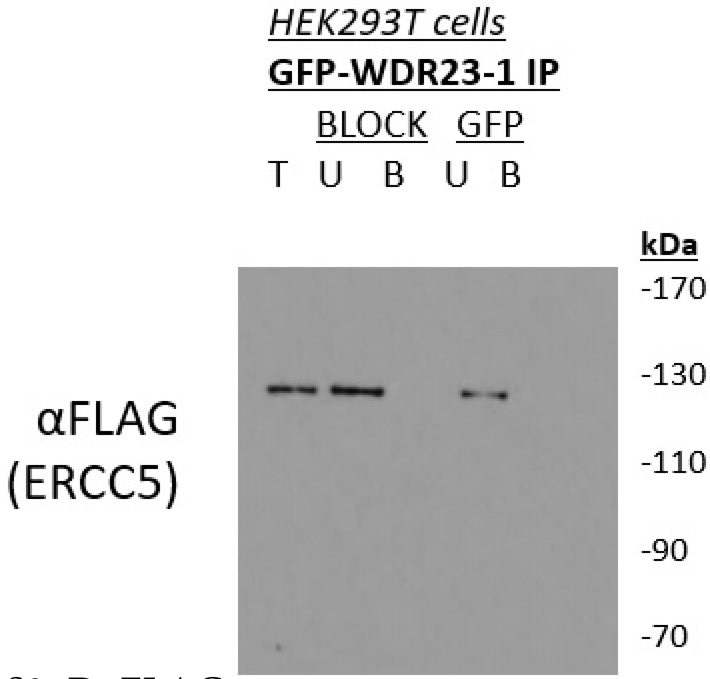
HEK293T cells
GFP-WDR23-1 IP
BLOCK
GFP
T
U
B
U
B
kDa
-170
-130
-110
-90
-70
αFLAG
(ERCC5)

fig_S2_D_FLAG

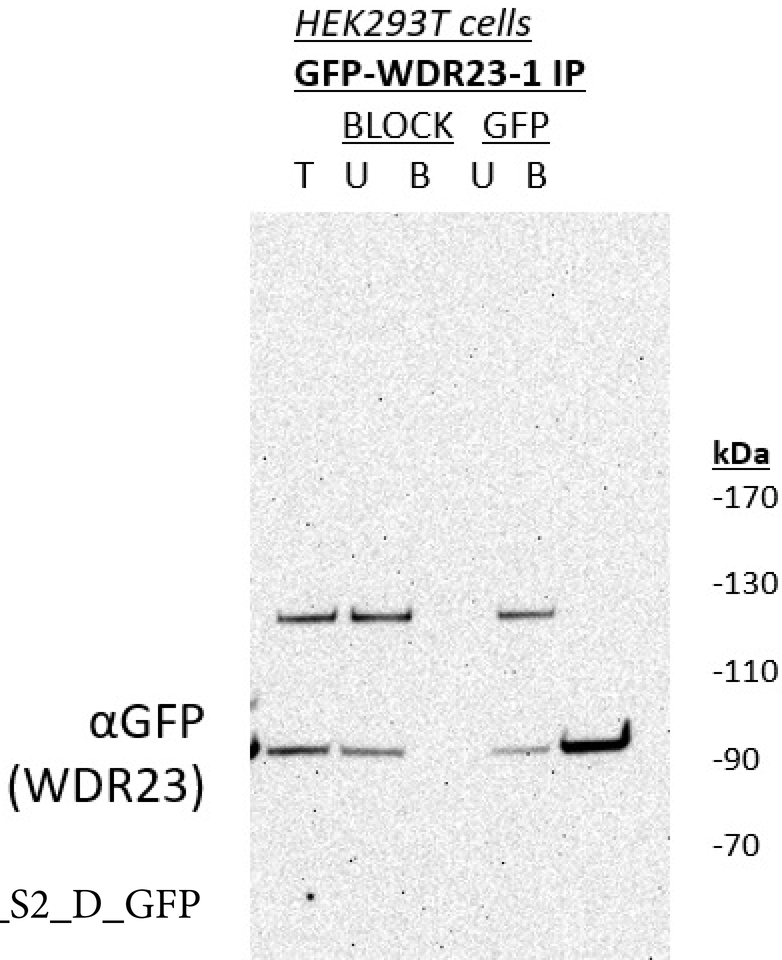
HEK293T cells
GFP-WDR23-1 IP
BLOCK
GFP
T
U
B
U
B
kDa
-170
-130
-110
-90
-70
αGFP
(WDR23)

fig_S2_D_GFP

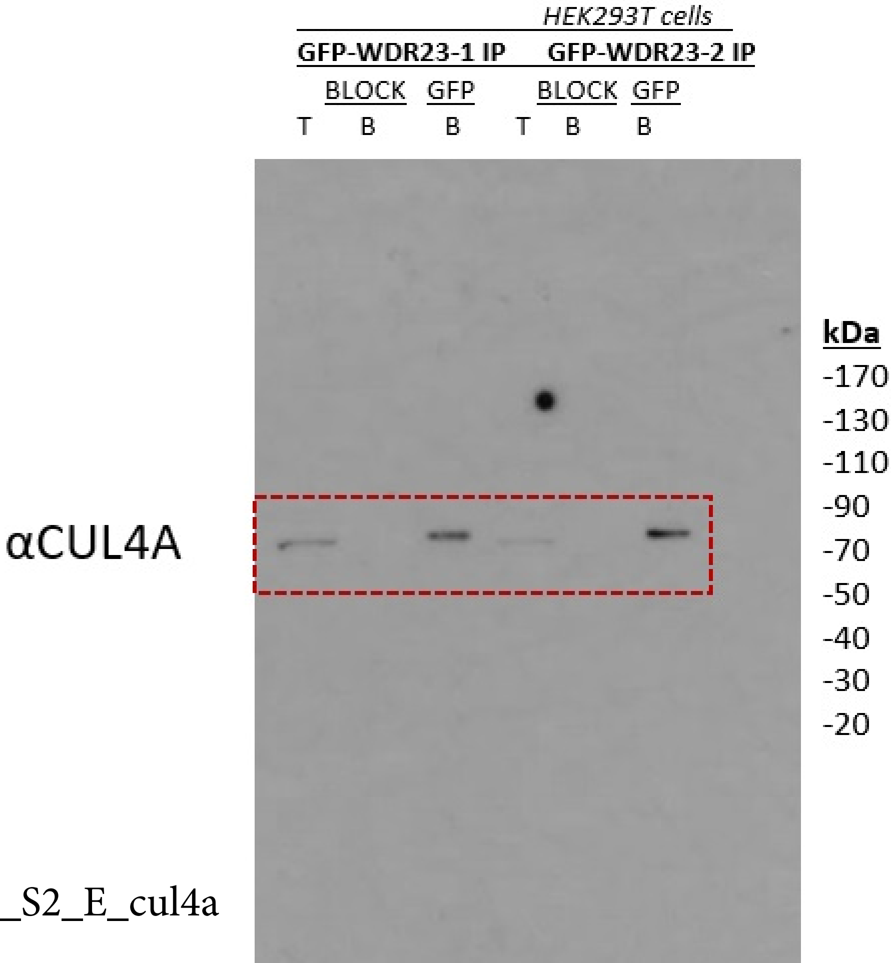

fig_S2_E_cul4a

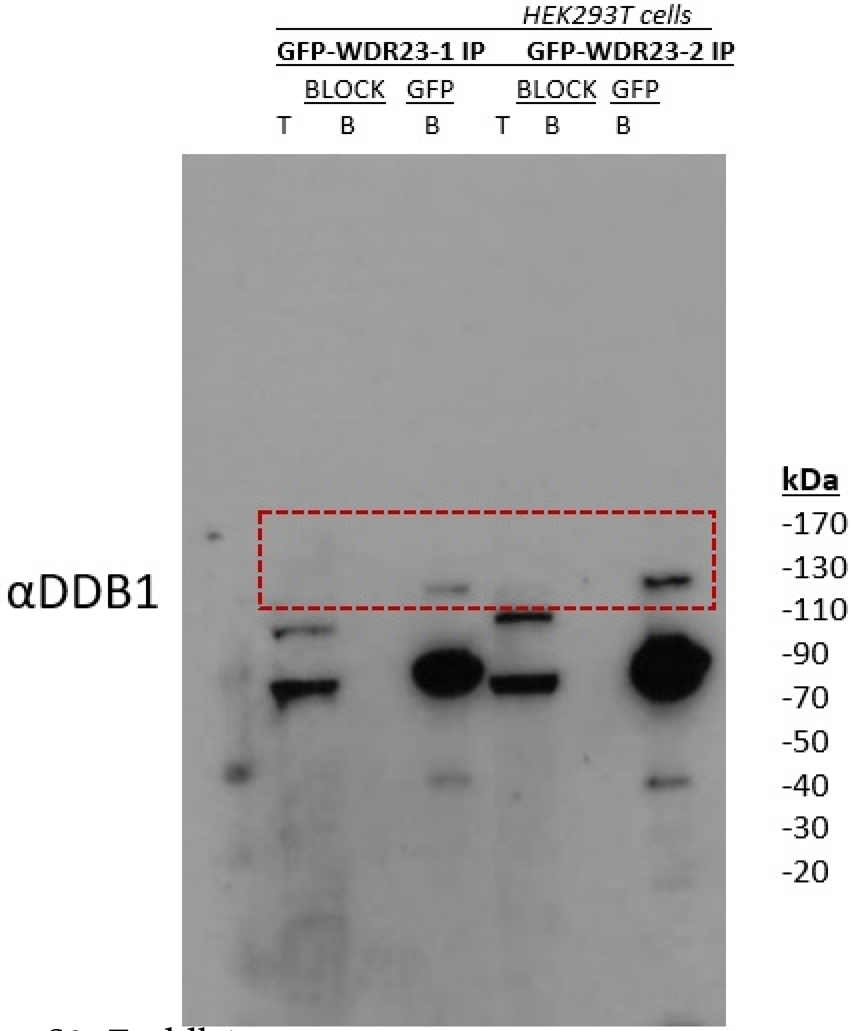
HEK293T cells
GFP-WDR23-1 IP
GFP-WDR23-2 IP
BLOCK
GFP
BLOCK
GFP
T
B
B
T
B
B
αDDB1
kDa
-170
-130
-110
-90
-70
-50
-40
-30
-20

fig_S2_E_ddb1

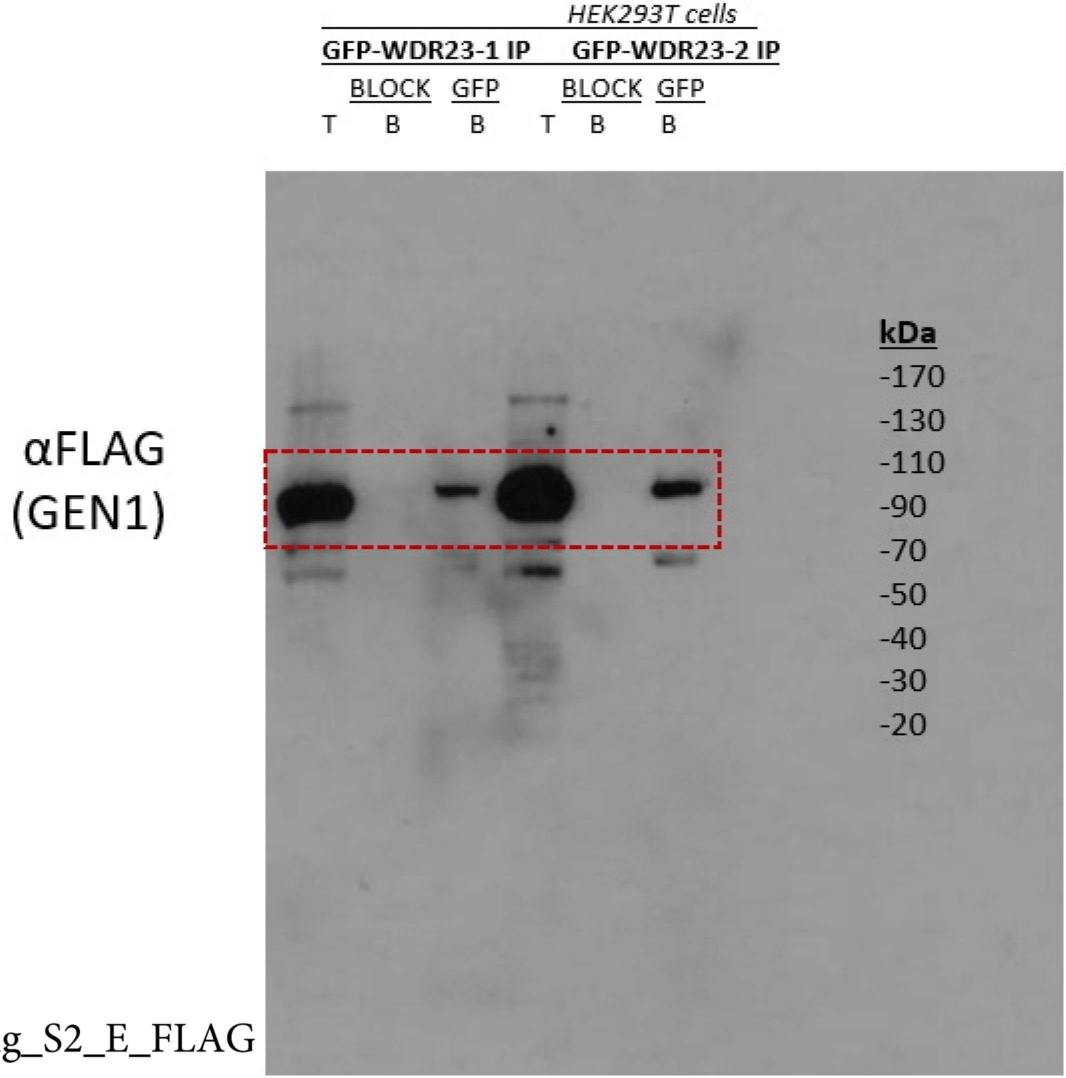
HEK293T cells
GFP-WDR23-1 IP
GFP-WDR23-2 IP
BLOCK
GFP
BLOCK
GFP
T
B
B
T
B
B
αFLAG
(GEN1)
kDa
-170
-130
-110
-90
-70
-50
-40
-30
-20

fig_S2_E_FLAG

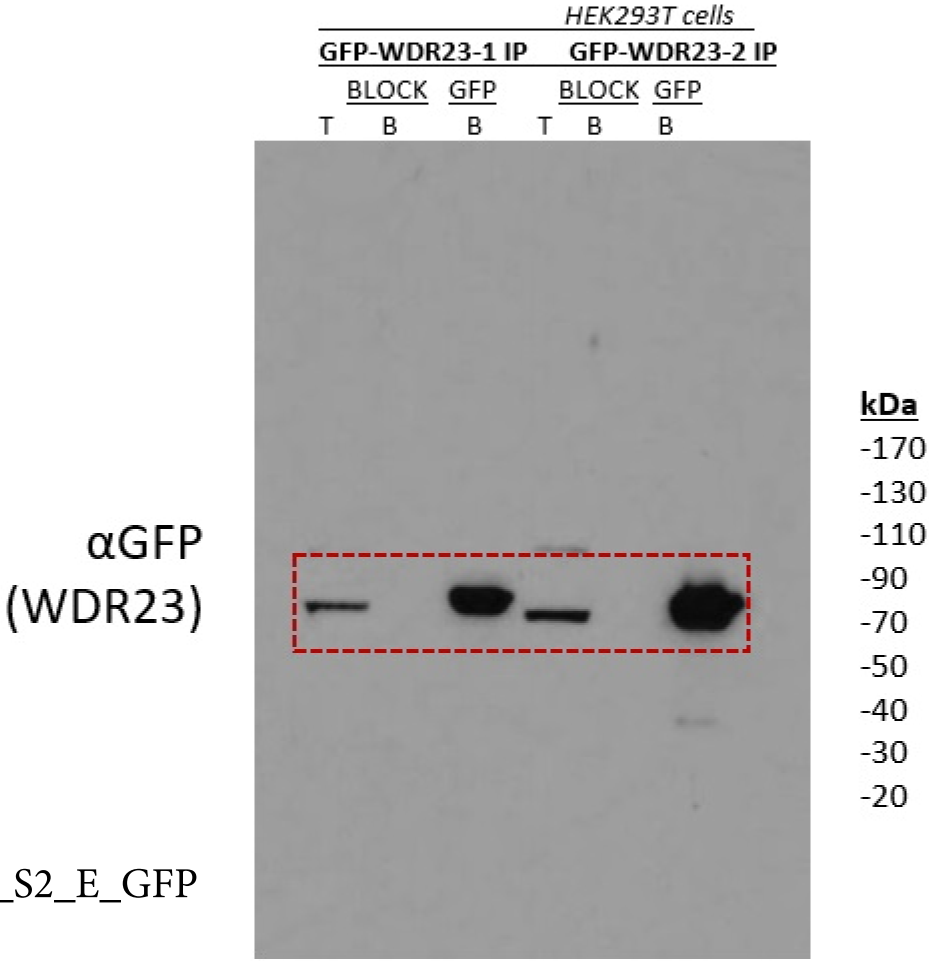
HEK293T cells
GFP-WDR23-1 IP
GFP-WDR23-2 IP
BLOCK
GFP
BLOCK
GFP
T
B
B
T
B
B
αGFP
(WDR23)
kDa
-170
-130
-110
-90
-70
-50
-40
-30
-20

fig_S2_E_GFP

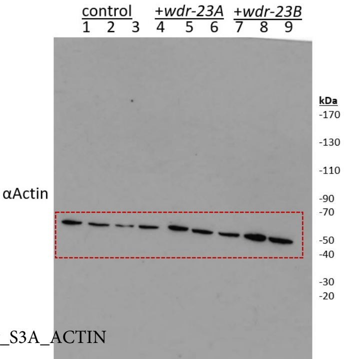

Fig_S3A_ACTIN

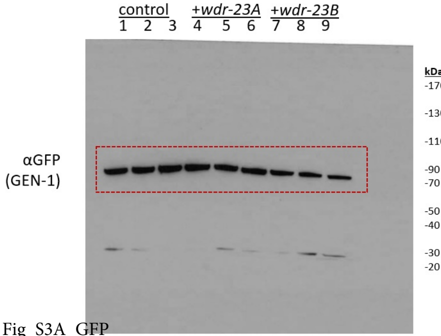
control
1 2 3
+wdr-23A
4 5 6
+wdr-23B
7 8 9
αGFP
(GEN-1)
kDa
-170
-130
-110
-90
-70
-50
-40
-30
-20
Fig_S3A_GFP

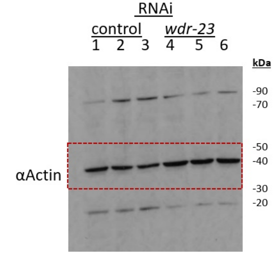

Fig_S3B_ACTIN

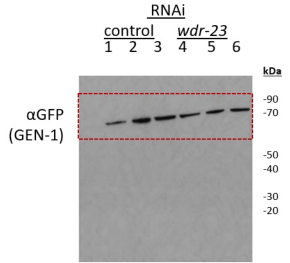

Fig_S3B_GFP

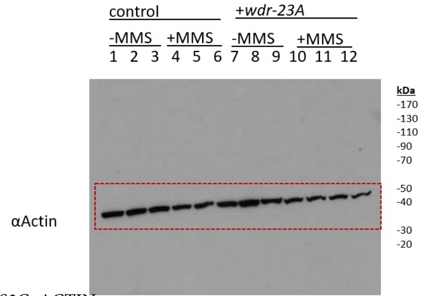

Fig_S3C_ACTIN

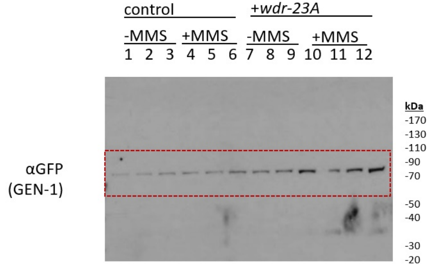
control
+wdr-23A
-MMS
+MMS
-MMS
+MMS
1 2 3 4 5 6 7 8 9 10 11 12
αGFP
(GEN-1)
kDa
-170
-130
-110
-90
-70
-50
-40
-30
-20

Fig_S3C_GFP

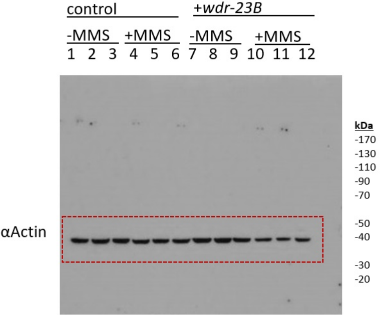

Fig_S3D_ACTIN

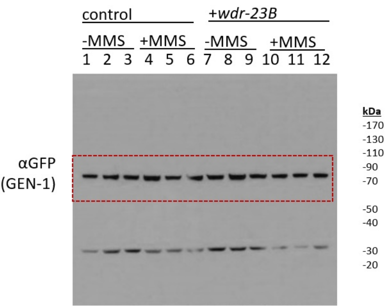
control
+wdr-23B
-MMS
+MMS
-MMS
+MMS
1 2 3 4 5 6 7 8 9 10 11 12
αGFP
(GEN-1)
kDa
-170
-130
-110
-90
-70
-50
-40
-30
-20

Fig_S3D_GFP

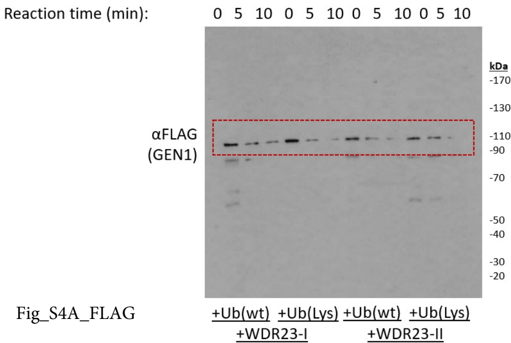
Reaction time (min):
0 5 10 0 5 10 0 5 10 0 5 10
αFLAG
(GEN1)
kDa
-170
-130
-110
-90
-70
-50
-40
-30
-20
+Ub(wt) +Ub(Lys) +Ub(wt) +Ub(Lys)
+WDR23-I
+WDR23-II

Fig_S4A_FLAG

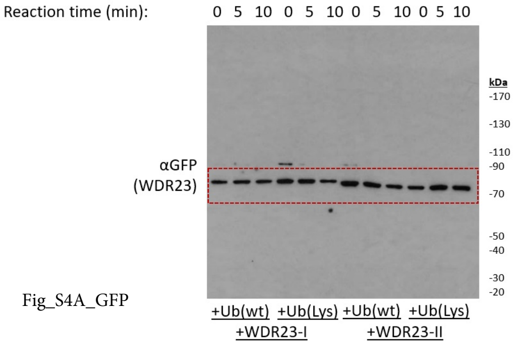

Reaction time (min):
0 5 10 0 5 10 0 5 10 0 5 10
kDa
-170
-130
-110
-90
-70
-50
-40
-30
-20
αGFP
(WDR23)
+Ub(wt) +Ub(Lys) +Ub(wt) +Ub(Lys)
+WDR23-I +WDR23-II

Fig_S4A_GFP

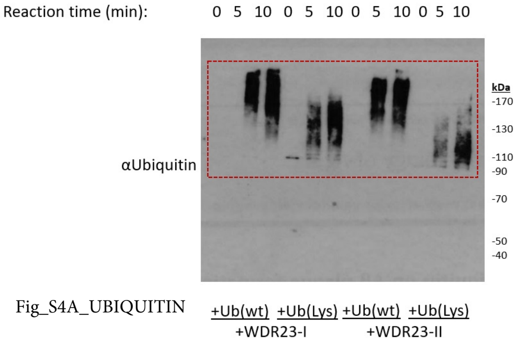

Fig_S4A_UBIQUITIN
