# Supplementary material for: Nuclear and cytoplasmic WDR-23 isoforms mediate differential effects on GEN-1 and SKN-1 substrates
Source: Sci Rep. 2019 Aug 13;9:11783. doi: 10.1038/s41598-019-48286-y (PMC6692315; doi:10.1038/s41598-019-48286-y)
Supplement: Supplementary file 3 — Supplementary Figures [file 41598_2019_48286_MOESM3_ESM.pdf]

Nuclear and cytoplasmic WDR-23 isoforms mediate differential effects on GEN-1 and SKN-1 substrates

Brett N. Spatola[1,2], Jacqueline Y. Lo[1,2,3], Bin Wang[4], and Sean P. Curran[1,2,5,*]

Supplementary Figure Legends
Supplementary Table 2
Supplementary Figures

SUPPLEMENTAL FIGURE LEGENDS

Supplemental Figure 1. Nuclear WDR-23B negatively impacts SKN-1 activity. (A) Representative images of *gst-4p::gfp* expression in control (*gst-4p::gfp*), *wdr-23(tm1817)*, *+wdr-23(A); wdr-23(tm1817)*, *+wdr-23(B); wdr-23(tm1817),* and gDNA rescue of both WDR-23A and WDR-23B *in wdr-23(tm1817)* animals. (B) Quantification from Fig. S1A depicting representative images of *gst-4p::gfp* expressed in control (*gst-4p::gfp*), *wdr-23(tm1817)*, *+wdr-23(A); wdr-23(tm1817)*, *+wdr-23(B); wdr-23(tm1817), and* gDNA rescue of both WDR-23A and WDR-23B *in wdr-23(tm1817)* animals. Images were taken at 20ms in L4 or later worms. Images were quantified using ImageJ and normalized to body size. n=5 animals for each strain. Data are mean ± s.e.m.; one-way ANOVA with multiple comparisons. n.s.=not significant, ****p<0.0001 (Table S1).

Supplemental Figure 2. WDR-23 isoforms interact with and differentially ubiquitinate GEN-1. (A) Reciprocal FLAG IP confirming both isoforms of WDR-23 IP with GEN-1 in HEK293T cells overexpressing tagged worm versions of GEN-1-FLAG,GFP-WDR-23, or GFP-(empty vector). (B) Reciprocal FLAG IP confirming both isoforms of WDR23 IP with GEN1 in HEK293T cells overexpressing tagged human versions of FLAG-GEN1 and GFP-WDR23. (C) GFP IP confirming specificity of interaction with GFP-(empty vector) and FLAG-GEN1. HEK293T cells were transfected with GFP-(empty vector) and FLAG-GEN1. (D) ERCC5 does not IP with WDR23 in HEK293T cells overexpressing tagged human versions of FLAG-ERCC5 and GFP-WDR23. (E) GEN1 IPs with WDR23, CUL4A, and DDB1 in HEK293T cells overexpressing tagged human versions of FLAG-GEN1 and GFP-WDR23. Block=blocked magnetic agarose GFP beads. GFP= magnetic agarose GFP beads. Input=2.5% of whole-cell extract used for IP . U=unbound/flow through fraction. B=bound fraction. αGFP probed for GFP-(empty vector)/GFP-WDR-23/GFP-WDR23 isoforms. αFLAG for GEN-1-FLAG/FLAG-GEN1/FLAG-ERCC5.

Supplemental Figure 3. Increased GEN-1 protein in response to DNA damage in cytoplasmic WDR-23A rescue worms. (A) Western blot image showing a slight but insignificant increase in GEN-1 protein in *+wdr-23A;gen-1::gfp* compared to control (*gen-1::gfp*) and *+wdr-23B;gen-1::gfp* animals. ~50 L4 worms were lysed per lane. n=3 independent biological replicates for each strain. (B) Western blot image showing no change in GEN-1 protein after RNAi depletion of *wdr-23* in animals expressing *gen-1::gfp*. ~50 L4 worms were lysed per lane. n=3 independent biological replicates for each condition. Western blot images of (C) *+wdr-23(A);gen-1::gfp* or (D) *+wdr-23(B);gen-1::gfp* in combination with control *gen-1::gfp* animals before and after exposure to 0.01% MMS. ~50 L4 worms were lysed per lane. n=3 independent biological replicates for each condition. αGFP probed for GEN-1::GFP. αActin probed for actin as a loading control.

.

Supplemental Figure 4. WDR-23 isoforms differentially ubiquitinate GEN-1. (A) Human ubiquitin assay depicting the size difference of modified GEN1 between WDR23 isoforms in the presence of the no K ubiquitin mutant. Immunoprecipitation samples of GFP-WDR23 isoforms and FLAG-GEN1 were incubated in ubiquitin reaction mixtures. Reaction time-points include 0-, 5-, and 10-minutes of incubation in 37ºC. (B) Quantification of unmodified GEN-1-FLAG in the presence of either isoform of GFP-WDR-

23 and WT or no K ubiquitin mutant over the 10-minute time-course (Fig. 4C). Isoforms of GFP-WDR-23 were used as loading controls. See methods for details on the quantification and Table S1 for stats. αGFP probed for GFP-WDR23 isoforms. αFLAG for FLAG-GEN1. αUbiquitin for ubiquitinated GEN1.

**Table S2. Top 5 BLASTP matches of sequence similarity to *C. elegans* GEN-1 (adapted from www.wormbase.org).**				
**BLASTP e-value**	**Species**	**Hit**	**Description**	**% Length***
5.3e-25	*S. cerevisiae*	SGD:YGR258C (RAD2)	Single-stranded DNA endonuclease; cleaves single-stranded DNA during nucleotide excision repair to excise damaged DNA; subunit of Nucleotide Excision Repair Factor 3 (NEF3); homolog of human XPG protein	82.7
6.5e-23	*S. pombe*	SW:P28706 (RAD13)	DNA repair protein rad13	82.9
6.6e-22	*D. melanogaster*	FLYBASE: CG10890 (MUS201)	Flybase gene name is mus201-PD	73.5
8.8E-24	*H. sapiens*	ENSEMBL: ENSP00000347978 (ERCC5)	Isoform 1 of DNA repair protein complementing XP-G cells	77.4
1.1e-13	*H. sapiens*	GEN1	Flap endonuclease GEN1 homolog 1	N/A
*% Length compared to C. elegans represents the extent of coverage of all matches on the target sequence.				

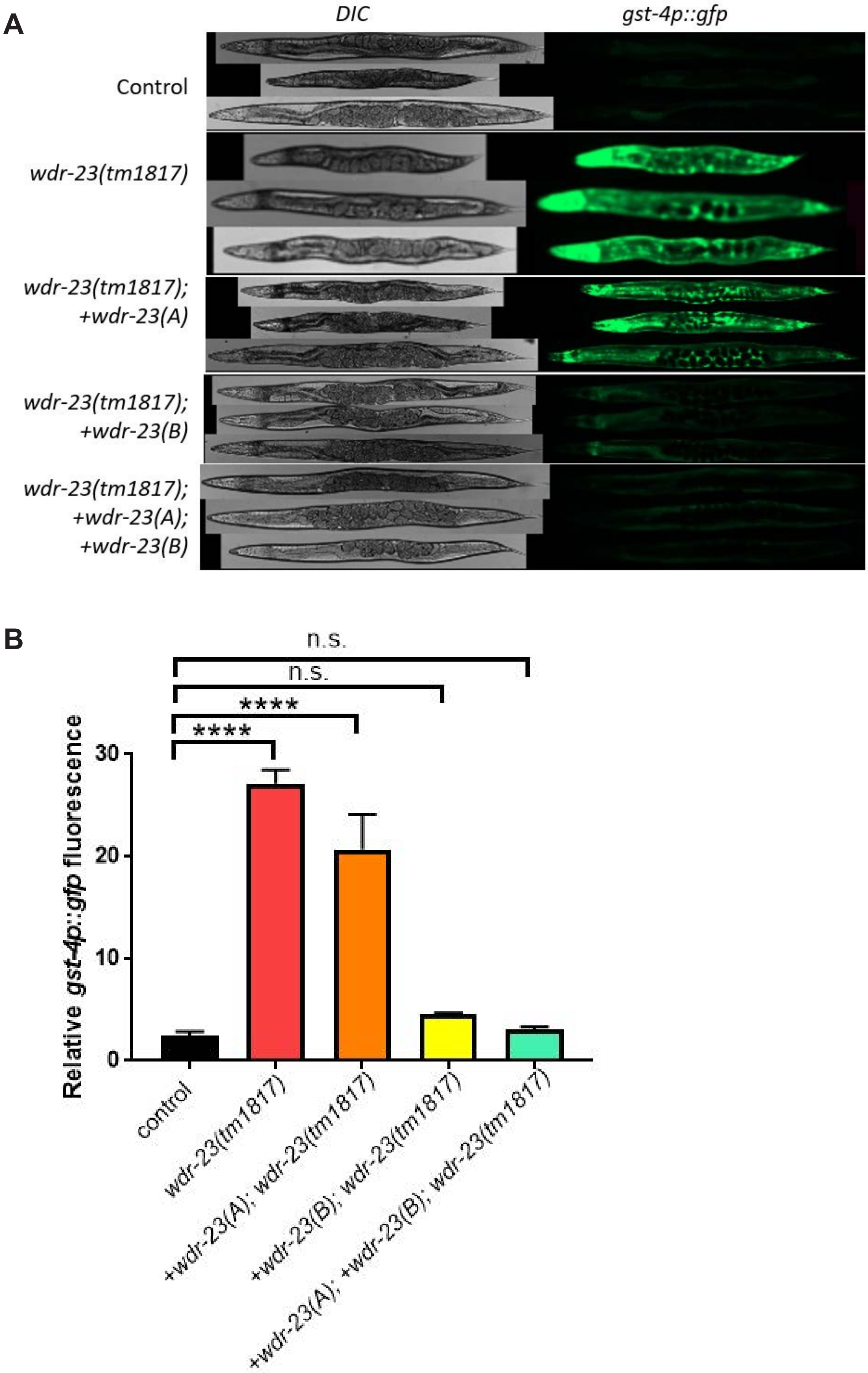

Spatola Supplemental Figure S1

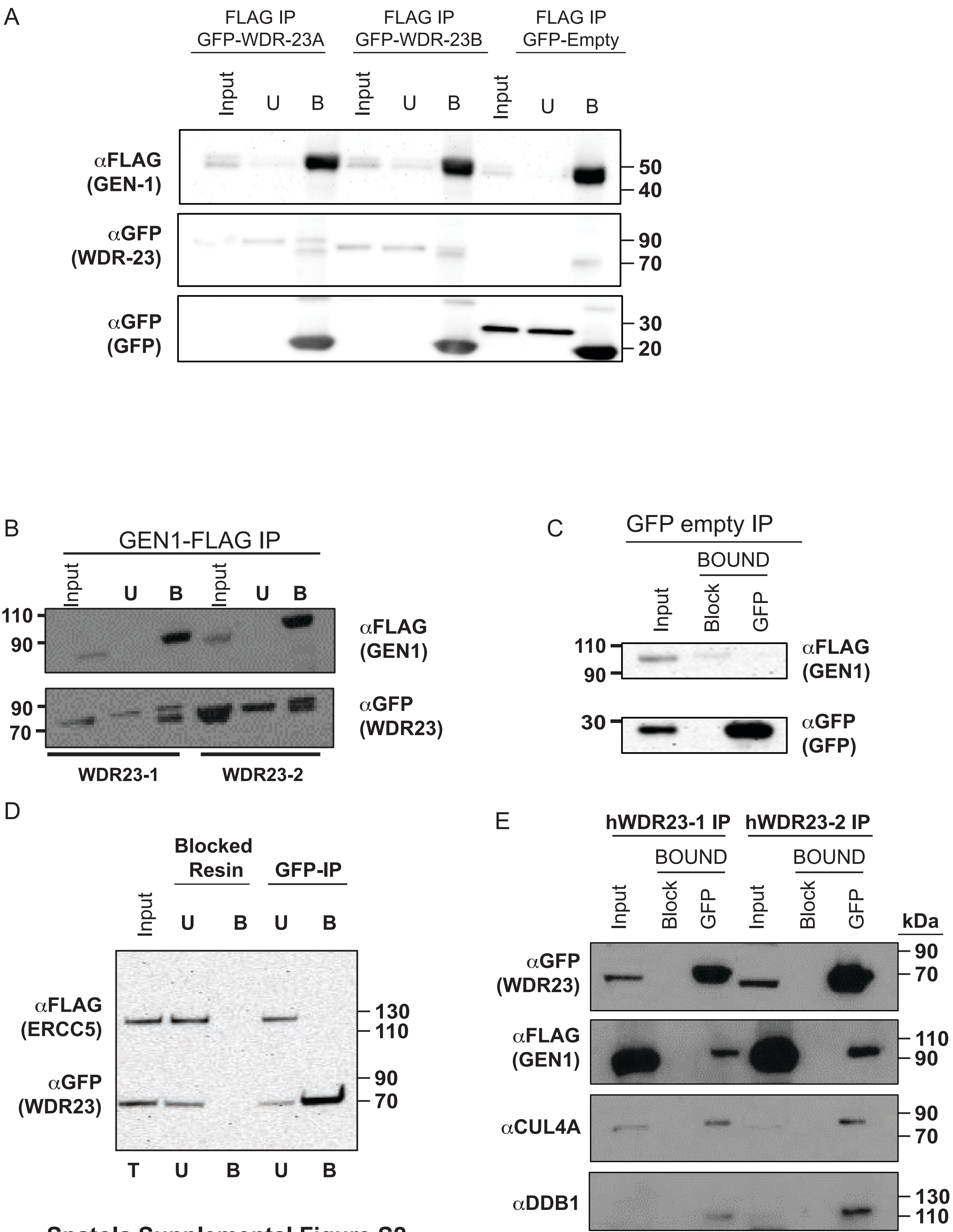

Spatola Supplemental Figure S2

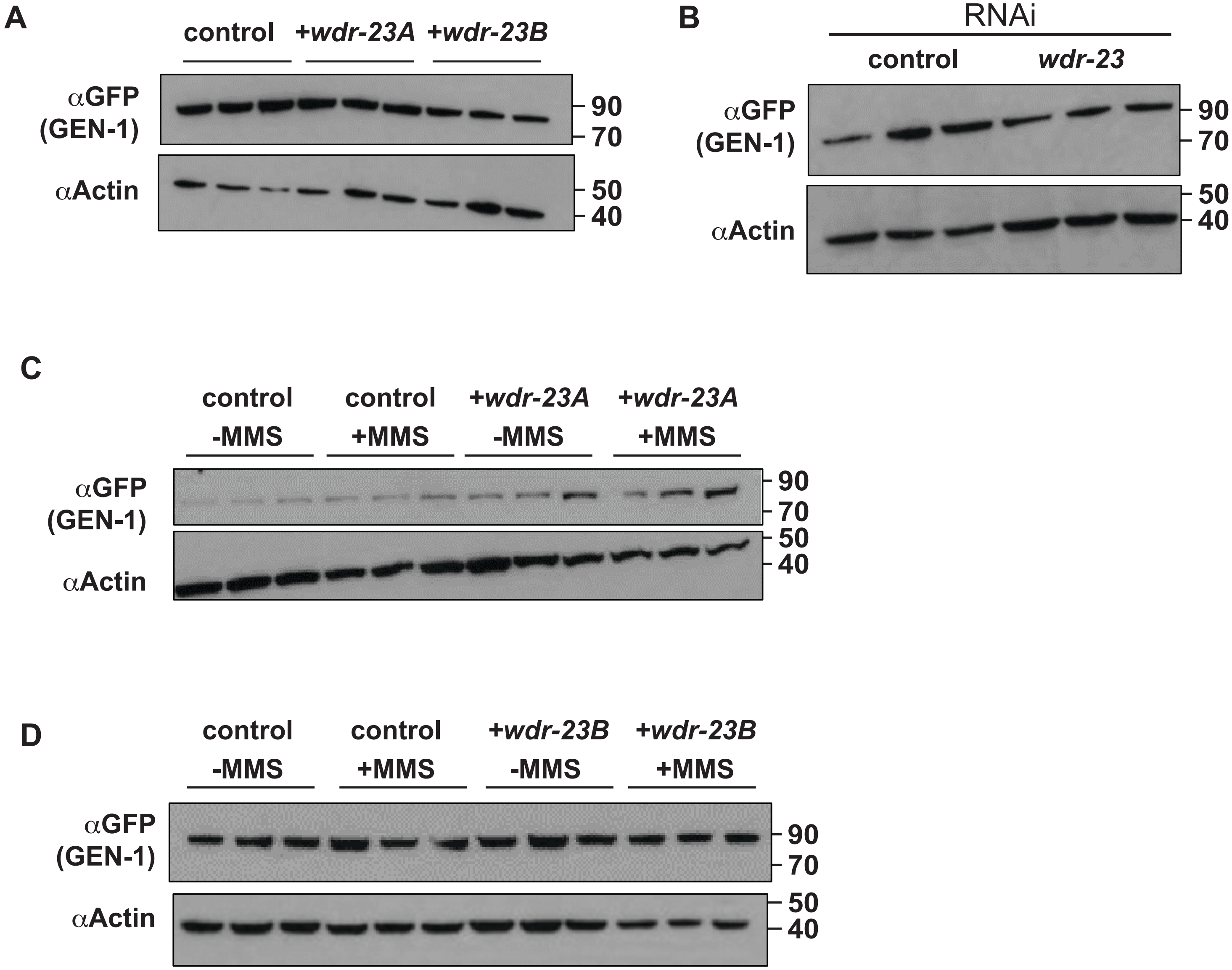

Spatola Supplemental Figure S3

A

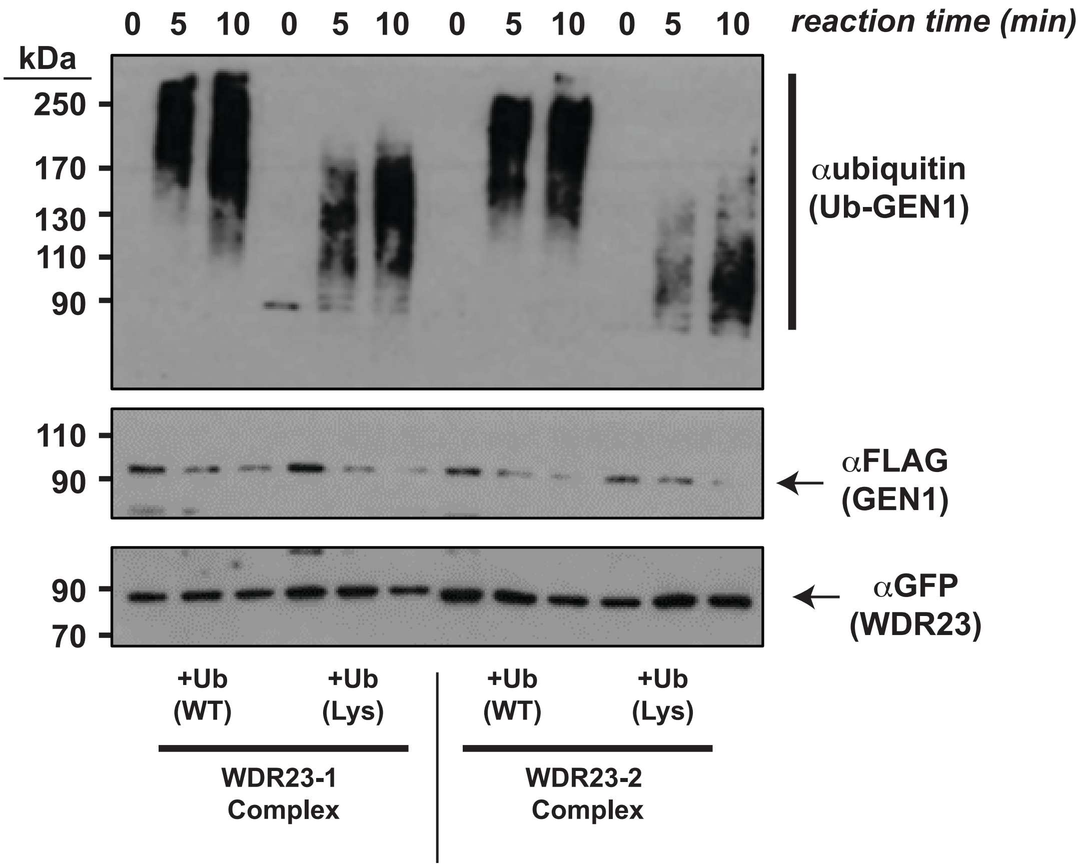

B

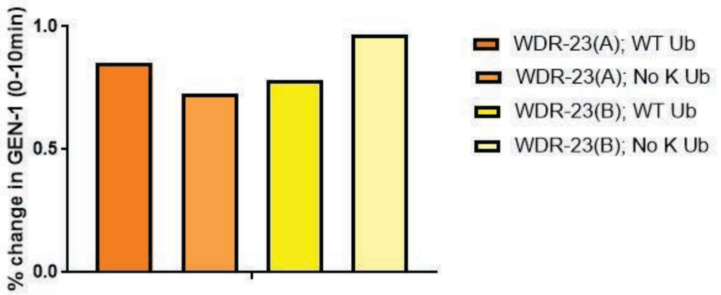

Spatola Supplemental Figure S4
